# GSTM3 and GSTP1: novel players driving tumor progression in cervical cancer

**DOI:** 10.18632/oncotarget.24796

**Published:** 2018-04-24

**Authors:** Alberto Checa-Rojas, Luis Fernando Delgadillo-Silva, Martín del Castillo Velasco-Herrera, Andrés Andrade-Domínguez, Jeovanis Gil, Orlando Santillán, Luis Lozano, Alfredo Toledo-Leyva, Alberto Ramírez-Torres, Patricia Talamas-Rohana, Sergio Encarnación-Guevara

**Affiliations:** ^1^ Laboratorio de Proteómica, Centro de Ciencias Genómicas. Universidad Nacional Autónoma de México, Cuernavaca, Morelos, México; ^2^ Programa de Genómica Evolutiva, Centro de Ciencias Genómicas, Universidad Nacional Autónoma de México, Cuernavaca, Morelos, México; ^3^ Departamento de Infectómica y Patogénesis Molecular, Centro de Investigación y de Estudios Avanzados del Instituto Politécnico Nacional, Av. Instituto Politécnico Nacional 2508, Col. San Pedro Zacatenco, Delegación Gustavo A. Madero, México

**Keywords:** GSTP1, GSTM3, cervical cancer, morpholinos, targets genes

## Abstract

The molecular processes and proteomic markers leading to tumor progression (TP) in cervical cancer (CC) are either unknown or only partially understood. TP affects metabolic and regulatory mechanisms that can be identified as proteomic changes. To identify which proteins are differentially expressed and to understand the mechanisms of cancer progression, we analyzed the dynamics of the tumor proteome in CC cell lines. This analysis revealed two proteins that are up-regulated during TP, GSTM3 and GSTP1. These proteins are involved in cell maintenance, cell survival and the cellular stress response via the NF-κB and MAP kinase pathways during TP. Furthermore, GSTM3 and GSTP1 knockdown showed that evasion of apoptosis was affected, and tumor proliferation was significantly reduced. Our data indicate the critical role of GST proteins in the regulation and progression of cervical cancer cells. Hence, we suggest GSTM3 and GSTP1 as novel biomarkers and potential therapeutic targets for treating cervical cancer.

**Significance:**

CC is particularly hazardous in the advanced stages, and there are few therapeutic strategies specifically targeting these stages. We performed analyses on CC tumor proteome dynamics and identified GSTM3 and GSTP1 as novel potential therapeutic targets. Knockdown of these proteins showed that they are involved in cell survival, cell proliferation and cellular evasion of apoptosis.

## INTRODUCTION

Cervical cancer (CC) is still the second most common cancer-related death in women worldwide, although it is in theory a preventable disease [1]. While the CC incidence in many countries decreased following the introduction of cytology screening [2], there are many countries in which this cancer remains a public health problem [3]. Unfortunately, the current treatment regimens for cervical cancer have shown limited survival benefits when used in advanced stages. The most common treatment includes a concurrent cisplatin-based chemoradiation therapy. Although, this treatment is regularly the only option for treating advanced stage cancers, in most cases it fails to fully eradicate the disease [4, 5]. Moreover, approximately 30% of patients experience lymph node recurrence and distant metastasis after primary treatment [6, 7]. The resistance to drugs and chemotherapy is also a major problem facing current cancer research [8]. Target gene-based strategies in cervical cancer remain promising; however, the research efforts of these therapies are aimed at prevention or early stages [9, 10]. In this sense, specific target gene-based strategies for late-stages or tumor progression are still urgently needed in the clinical setting [11].

Tumor progression (TP) involves metabolic changes and dysregulation of cellular processes that results in the pathological progression of the disease [12]. The tumor proteome represents a particular metabolic stage and is a dynamic entity that varies during TP. Therefore, we believe that studying the proteome dynamics in CC, would be highly valuable to understanding TP and the progression of the disease [13, 14]. During TP, changes in the proteome of cancer cells favor growth and interrupt host homeostasis. Measuring these proteomic changes will help elucidate the interplay between pathological processes and early events that lead to cancer progression [13–15].

In several cancers, members of the glutathione S-transferase (GST) family have been reported as being overexpressed and in most cases have been linked to poor prognosis and chemoresistance [16–19]. The GSTs are a family of enzymes that exhibit diverse functions, including detoxification of xenobiotic compounds, immune system evasion and apoptosis inhibition [20]. Particularly, GSTP1 and GSTM3 have been reported as being dysregulated in cancers such as: prostate cancer [21], triple-negative breast cancer [22], lung cancer [23], and colorectal cancer [17, 24]. GSTP1 plays a regulatory role by interacting with TRAF2 and decreasing signal transduction from receptors in the TNF-α and JNK kinase pathways, which are responsible for apoptosis activation [25, 26]. The protein expression of GSTM3 has been analyzed in colon cancer, and its overexpression is considered a marker of regional lymph node metastasis [17]. On the other hand, subexpression of GSTM3 is associated with better survival in urinary bladder cancer [27].

The proteome dynamics of TP in CC have been poorly explored and understood. In this study, we used xenografts of CC cell lines to analyze, through a proteomic approach, the protein expression differences during TP. We found GSTP1 and GSTM3 among the proteins that consistently increased their levels during tumor growth. We further explored the critical role of these proteins for cell survival and tumor progression through their knock-down. In addition, we correlated the abundance of these protein levels, in CC biopsies, with patient survival. Therefore, we believe at least these two members of the GST family could be used for prognosis purposes, and they could potentially be excellent candidates for target gene-based therapies for CC.

## RESULTS

### Tumor progression model

**Figure 1:**
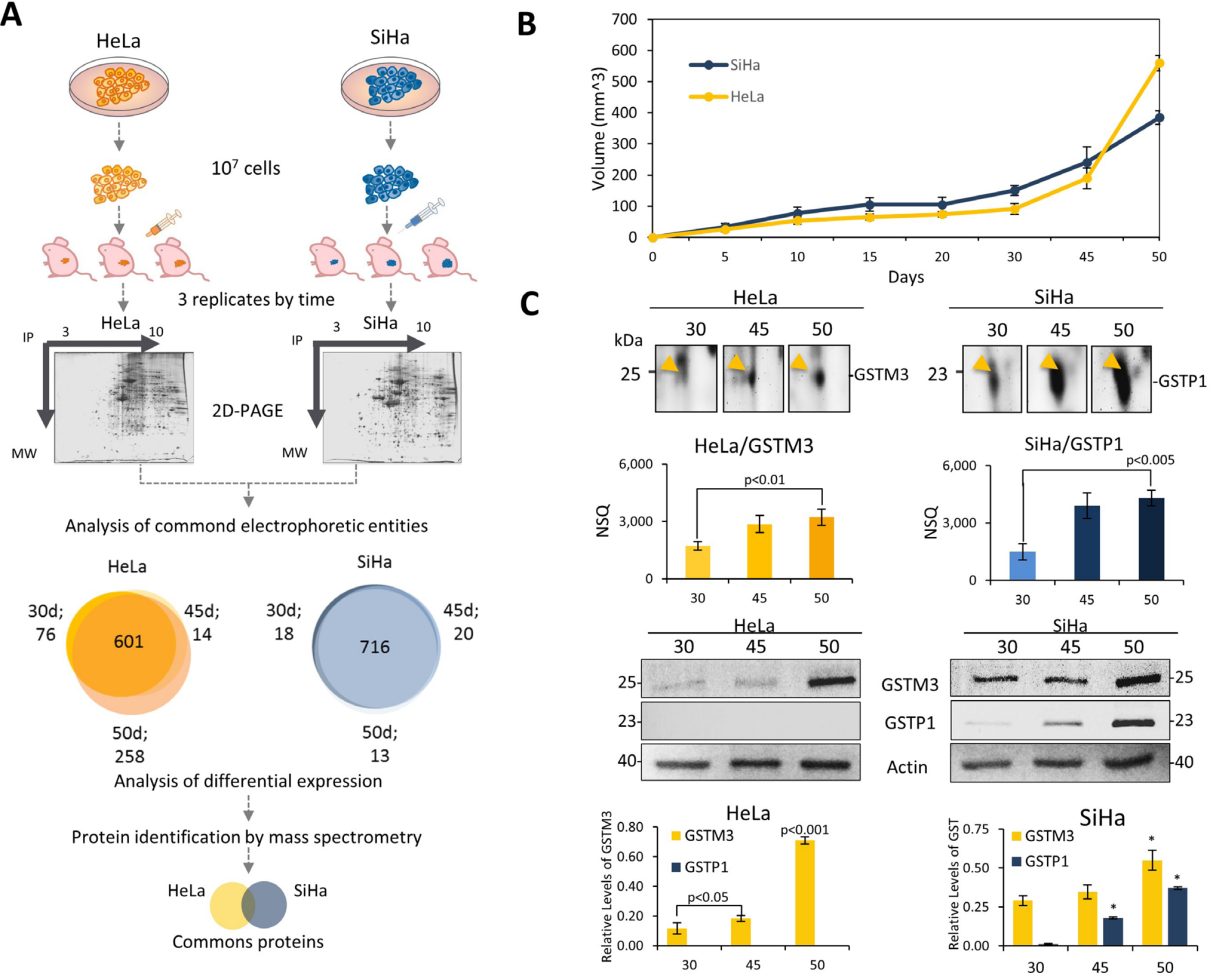
Proteomics analysis of tumor progression model of xenotransplanted cervical cancer cell lines revealed proteins involved in the tumor growth (**A**) Experimental design to study the proteome dynamics of tumors xenotransplanted in a murine model. HeLa and SiHa cell lines were cultured to 70% of confluence and 10^7^ were injected in female Nu/Nu mice. Tumor proteins were extracted and analyzed by 2D-PAGE. The spots of three independent tumors (biological replicates) were compared and analyzed. An analysis of common electrophoretic entities was employed. Electrophoretic entities shared between HeLa tumors, and SiHa tumors are represented in a Venn diagram. The proteomic profile was obtained from each time and then compared to find differentiated proteins during TP. (**B**) Kinetic curves of tumor growth HeLa tumors (yellow) and SiHa tumors (blue). (**C**) 2D SDS-PAGE region and expression analysis of GST during TP. Regions of 2D-SDS-PAGE of GST proteins shows an increase over time. Protein expression levels were obtained by normalized spot quantity (NSQ), and western blot values were quantified using β-actin protein as an internal control. GSTM3 (HeLa, yellow line) and GSTP1 (SiHa, blue line). Representative results of western blotting of GSTM3, and GSTP1 show that, HeLa only expressed GSTM3 and SiHa tumors expressed both GSTM3 and GSTP1. Samples were mixed (1:1), cell lysates were subjected to western blot with 20 µg of protein per sample. All assays were performed in triplicate. The data are presented as means (± standard deviation, SD) of three independent experiments, *P*-value < 0.05 was considered statistically significant (^*^P-value < 0.01, with respect to 30 days).

In order to create a suitable model that allowed us to study TP, we used cervical cancer cell lines (SiHa and HeLa) to generate xenotransplanted tumors in mice. Cancer cells were cultured to 70% of confluence and 10^7^ cells were inoculated into female Nu/Nu mice. The tumor volume was measured according to the equation described in experimental section, at seven different times during tumor progression (5, 10, 15, 20, 30, 45, 50 days post-inoculation). The first four measurement times showed low progression of tumor growth. However, in the final points of the kinetics the tumor volume grew exponentially for HeLa cell tumors. From day 30 to day 45 the average tumor volume was duplicated, and by day 50 the average volume was 3-fold higher than the previous measurement. For SiHa cells the tumor growth rates were lower than those of HeLa. From 30 to 45 days, SiHa tumors grew 1.6-fold, while in the last five days the tumors were on average 1.6-fold larger (Figure 1A–1B). According to these findings we decided to further evaluate the dynamics of TP at proteome level between 30, 45 and 50 days post-inoculation.

### 2DE-PAGE and protein identification analyses

Tumors from both cancer cells were collected and total proteins were extracted and analyzed by means of 2DE-PAGE. Three replicates of the 2DE-PAGE analysis were performed for each cell type and for each time studied. The image analysis was carried out by using PDQuest software. On average, we were able to detect 866 spots for HeLa tumors samples in each replicate. For SiHa tumors, the average number of detected spots on 2DE-PAGE images for individual replicates was 766 entities. From the 2DE-PAGE maps, we determined the correlation coefficient between replicates from each tumor age and cell type. In all tumors, the correlation coefficient was greater than 0.7 for both cell types (Supplementary Table 1).

In addition, 601 spots were detected in the three stages of the progression on HeLa tumors, while the number of commonly detected spots in SiHa tumors was 716 (Figure 1A). For protein identification, a total of 90 gel spots from tumors including both cell types, were selected based on their abundance patterns between the ages of the tumors. All gel spots were processed as described in the experimental section and were identified following MALDI-TOF mass spectrometry analysis. From HeLa tumors, we identified 46 different proteins (Supplementary Table 2), including 34 with constant expression throughout TP, 7 proteins that were down-regulated throughout TP, 3 proteins that were up-regulated during tumor growth, and 2 proteins that had oscillating expression pattern. In tumors from SiHa cells, a total of 44 proteins were identified (Supplementary Table 3). The identified proteins were distributed according to their expression pattern, with 20 that had no differences in the three evaluated tumor ages, 8 that had decreased abundance during TP, and 16 that showed an increased abundance. Analyzing all identified proteins in tumors from both cell types, we found that 34 proteins were shared between the two tumor types, including 14 that showed the same expression pattern (Supplementary Figure 1, Supplementary Tables 2–3). Among the proteins with patterns showing an increasing trend, we identified two members of the glutathione S-transferase family (GSTM3 and GSTP1). GSTM3 was identified in tumors from HeLa cells and GSTP1was identified in tumors from SiHa cells (Figure 1C). The expression levels of both proteins were confirmed by western blot analysis (Figure 1C). In addition, the western blot revealed that SiHa tumors showed similar expression patterns for both GSTs. However, the pattern was only confirmed for GSTM3 in HeLa tumors, while GSTP1 was not detectable in any tumor stage.

### Bioinformatics analysis

**Figure 2:**
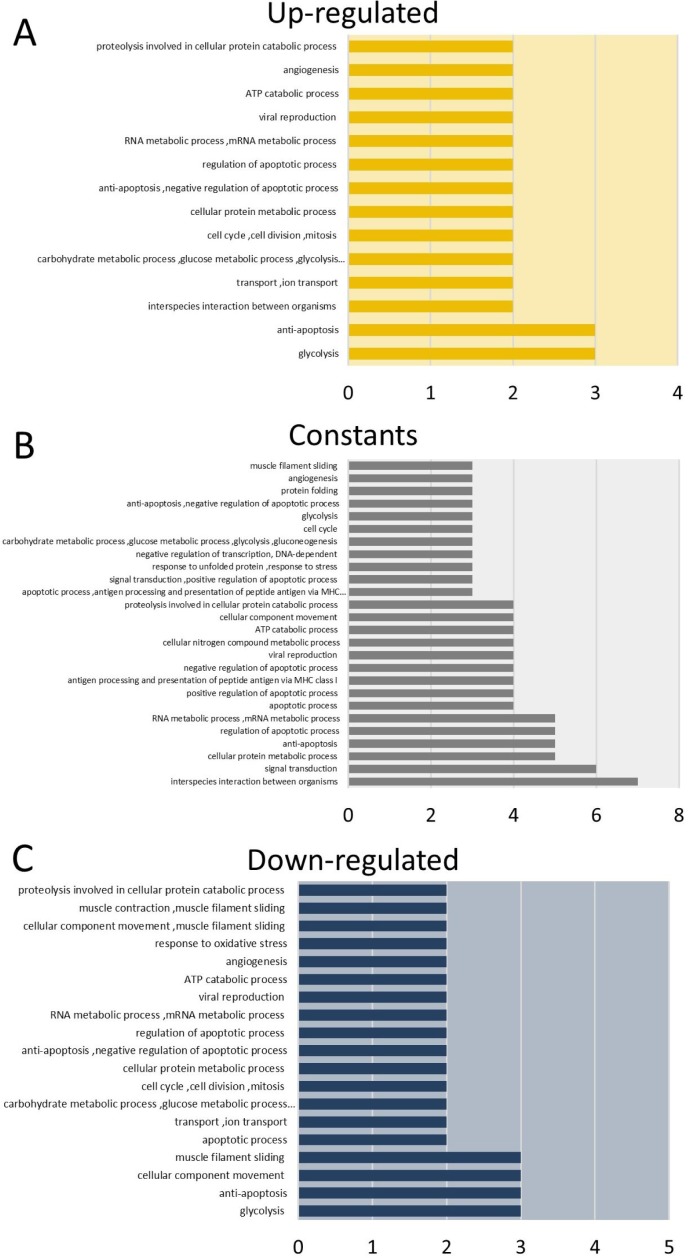
Gene ontology enrichment analysis of identified proteins in tumors from HeLa and SiHa cells (**A**) Biological processes enriched in Up-regulated shared proteins. (**B**) Biological processes enriched in constantly shared proteins. (**C**) Biological processes enriched in Down-regulated proteins. The analysis was performed using GeneCodis website.

**Figure 3:**
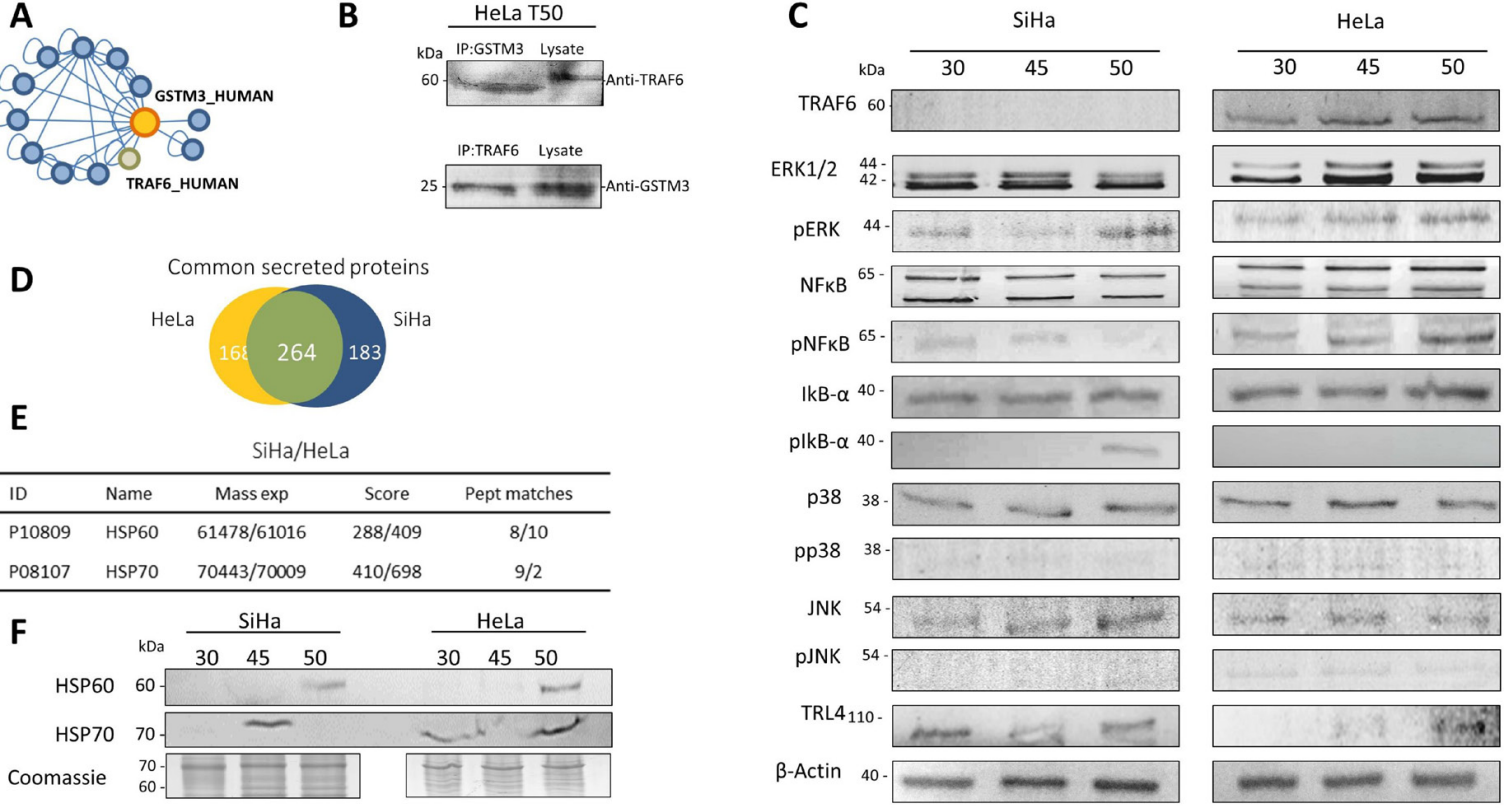
(**A**) Cytoscape interaction network representing GSTM3–prey interactions. GSTM3–prey interactions were visualized by network edges. This analysis was performed to obtain the protein-protein interactions reported by the SysBiomics databases, in which we observed that TRAF6 interact with GSTM3. (**B**) Co-immunoprecipitation of GSTM3 and TRAF6. (**C**) Western blotting was performed for TRAF6, ERK, p-ERK, pERK, NF-κB, pNF-κB, IKBα, pIKBα, p38, pp38, JNK, pJNK, and TLR4 in the protein extracts from tumors xenotransplants in female mice. (**D**) Proportional Venn diagram of secreted proteins of CC cell lines with 264 common proteins. (**E**) Two proteins were identified in secreted proteins that can activate the TLR4 signal-pathway expressed *in vitro* in HSP60 and HPS70 (**F**) Western blot of TRL4 activators HSP70 and HSP60 in the proteins secreted by CC tumors, HSP60 was expressed in SiHa and HeLa tumors at day 50, and HSP70 protein expressed in SiHa tumors at 43 days and Hela tumors at 30 and 50 days.

We used the proteins identified in tumors from both cell lines to perform a functional enrichment analysis based on Gene Ontology (GO) biological processes (Figure 2, Supplementary Table 4). The proteins were grouped according to their expression levels and subjected to the enrichment analysis. Our results indicated that the proteins with increased levels during TP are mainly involved in anti-apoptotic, cell division, glycolysis, angiogenesis, viral reproduction and regulation of apoptosis processes (Figure 2A). On the other hand, among the proteins whose expression is down-regulated during TP, the enriched biological processes were the regulation of apoptotic processes, antigen processing and the presentation of peptides via MHC class I, protein polyubiquitination and the response to unfolded proteins (Figure 2C). For proteins with constant expression, we found that they are involved in processes related to the regulation of apoptosis and glycolysis among others (Figure 2B). In addition, including all identified proteins, the results suggest that during TP over-represented pathways are related to the cellular response to stress, namely the MAPK6/MAPK4 and NIK/NF-κB signaling pathways (Supplementary Tables 4–5). On the other hand, the data mining analysis revealed that GSTM3 and GSTP1 interact with tumor necrosis factor receptor-associated factors (TRAFs) proteins. Specifically, the interaction of GSTP1 with TRAF2 was previously validated in HeLa cells [25], and GSTM3 was reported as interacting with TRAF6 [43] (Figure 3A).

### GSTM3 interacts with TRAF6 in CC tumors

To demonstrate that this interaction occurs under physiological conditions, first we demonstrated the expression of TRAF6 in both HeLa and SiHa tumors (Figure 3B). We observed that TRAF6 was only expressed in HeLa tumors. These results are consistent with other results reported, indicating that GSTP1 only interacts with TRAF2 [25]. Then, we developed the interaction analysis, using 50 day-old HeLa tumor lysates subjected to coimmunoprecipitation (IP) assay. We found that GSTM3 coimmunoprecipitates with TRAF6 and vice versa (Figure 3B). Therefore, GSTM3 associates with TRAF6 in CC tumors.

### Modulation of MAPK signaling during TP

Given the importance of TRAF proteins in over the downstream activation of the mitogen-activated protein kinase (MAPK) cascade, we performed a western blot analysis of CC TP. We detected phospho-NF-κB p65 and phosphorylated-extracellular signal-regulated kinase (ERK), pJNK, and pp38. Our results demonstrated that p38 and JNK phosphorylation were down-regulated throughout the progression of across the time in CC tumors but not in pNF-κB (ser529) or pERK (Figure 3C, Supplementary Table 6). These finding indicate that during TP the apoptotic processes are repressed and therefore cell proliferation is being constantly activated.

### Secreted endogenous activators of toll-like receptor 4 in cervical cancer

It is known that the activation of the TLR4 pathway is not only driven by the presence of lipopolysaccharides (LPS) from bacterial infections [44] but also by endogenous activators [45, 46]. To demonstrated that CC cell lines can express endogenous activators of toll-like receptor 4 (TLR4), we performed an *in vitro* analysis of secreted proteins using both the HeLa and SiHa cell lines (Supplementary Figure 2A, Supplementary Table 7-S8). The secreted proteins were analyzed by LC-MS/MS and a total of 432 HeLa and 447 SiHa proteins were identified, of which 264 were common to both cell lines (Figure 3D). Among the reported endogenous activators of the TLR4 pathways, we were able to identify two members of the heat shock proteins family, HSP60 and HSP70, that were secreted by both cell lines (Figure 3E). To determine whether these proteins were also expressed during TP, we then analyzed the *ex vivo* secreted proteins in CC tumors by western blot (Figure 3F, Supplementary Figure 2B). Our results, were similar in *in vivo* and *ex vivo* experiments, indicating that the secretion of HSP60 and HSP70 could activate TLR4 signaling.

### GSTM3 interacts with E7 from HPV18

**Figure 4:**
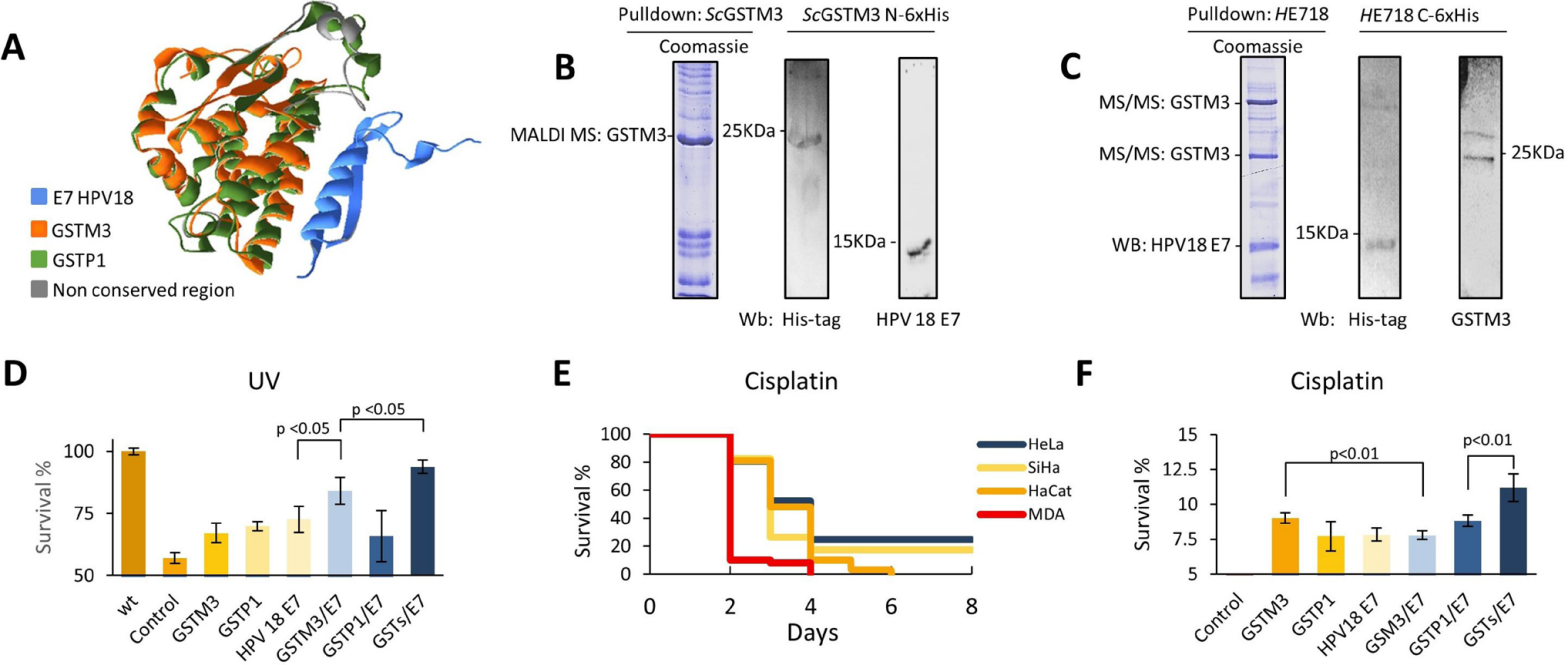
(**A**) Superposition of GSTP1 and GSTM3 shows high structural similarities (green-orange), non-conserved structures (gray), and HPV18 E7 (blue). (**B**) The interaction of human recombinant protein ScGSTM3 N-6x his-tag with E7 of HPV 18 protein. (**C**) HeLa cells were transfected with plasmids to express HE718 C-6x his-tag as indicated in the methods. Cell lysates were subjected to Ni-6x his tag pull-down and western blot with anti-His-Tag and specific antibodies. GSTM3 was identified by MALDI-TOF and/or MS/MS. (**D**) PAEP assays. Cells were exposed to UV (UVB for 15 seconds), subjected to exogenous protein complementation and allowed a 24-hour recovery period. The MDA cell line was used as a negative control for HPV, GSTM3, and GSTP1. (**E**) Survival assay with 6.0 mM of cisplatin. MDA cell line survived, until the fourth day (red), and HaCaT cell line survived until the sixth day (orange). HeLa and SiHa CC cell lines survived until the eighth day, which was the last day that this trial was analyzed. (**F**) PAEP assays, using the MDA cell line with 6.0 mM of cisplatin and a four days of recovery period. M3/P1/E7 with 10.7% survival. Assays were performed in triplicate. The data are presented as means (SD) of three independent experiments.

Mileo *et al.* previously demonstrated that GSTP1 interacts with E7 from HPV16 and that this interaction enhances the survival of cells [36]. Here we wondered if GSTM3 can interact with E7 from HPV18 in cells positive for infection with this HPV serotype. We performed a structural superposition alignment between the GSTP1 and GSTM3 proteins using the MAMMOTH program [47], and the used Swiss PDB Viewer software (Deep View) v4.1 to visualize the results (Figure 4A, Supplementary Table 10) [35]. The model of the GSTP1 protein docking with HPV16 E7, provided by the Mileo group, was used to perform our structural superposition. The alignment shows conserved and non-conserved regions by comparing the distances between alpha carbons and amino acid backbone sequences. The results suggest that the GSTM3 and HPV18 E7 proteins could interact, in a similar way to way GSTP1 interacts with E7 from HPV16 (Figure 4A).

To demonstrate this interaction, we generated a construct to express a recombinant human GSTM3 protein with a His-tag added at the N-terminus (N-6x His-tag) in *S. cerevisiae* (Supplementary Figure 3A). GSTM3 was identified through anti-His-tag western blotting and peptide mass fingerprinting (Figure 4B, Supplementary Figure 3A). After capturing recombinant GSTM3, it was incubated with a protein extract from HeLa cells (HPV18-positive) (Supplementary Figure 3B). The E7 protein from HPV18 co-eluted with GSTM3 N-6x-his-tag and was identified using a specific antibody by western blotting (Figure 4B). To verify this interaction, we generated an HPV18 E7 construct in *S. cerevisiae*, but we could not obtain a stable strain expressing the protein. We then generated a plasmid construct that expressed a recombinant HPV18 E7 C-6x-his-tagged protein in HeLa cell lines and performed a pull-down assay (Supplementary Figure 3C). Notably, our results demonstrated that GSTM3 can interact with HPV18 E7 (Figure 4C, Supplementary Figure 3D).

### GSTs and E7 from HPV18 contribute to cell survival

Once we had demonstrated the interaction of GSTM3 with E7 from HPV18 we assessed the relevance of this interaction for cell survival. We developed a UV stress sensitivity assay in an MDA-MB-231 cell line that was negative for HPV18, GSTM3 and GSTP1 [22] (Supplementary Figure 4A), using recombinant GST and HPV18 E7 to perform at phenotype analysis through exogenous protein complementation (PAEP). We demonstrated that, under UV stress (15 sec. UV; IC50), the GSTM3/HPV18 E7 cells exhibited an 84.1% survival rate, whereas the GSTP1/GSTM3/E7 cells exhibited a survival rate of 93.7% after a 24 h recovery period. These results could indicate that there is a synergistic effect between GSTs and viral proteins (Figure 4D). Given these observations, we performed an *in vitro* assay in which CC and negative cell lines were exposed to 6 mM cisplatin [37]. This concentration of cisplatin completely kills 100% of MDA-MB-231 by the fourth day of treatment. All HaCaT cells were killed in six days (Figure 4E). Surprisingly, the cell lines that co-expressed GSTs and HPV E7 survived for at least eight days post-treatment (SiHa 17% and HeLa 24% of confluence) (Figure 4E). To demonstrate that GST/HPV18 E7 interaction was responsible for this resistance, a PAEP assay was performed using the MDA-MB-231 cells including recombinant GSTM3, GSTP1, and HPV18 E7. The results confirmed that cell lines expressing members of the GST protein family and E7 from HPV have an advantage in terms of cell survival when treated with a xenobiotic agent (Figure 4E, 4F). We observed an increase in the survival of cells expressing any of these proteins (HPV18 E7, GSTM3 or GSTP1); nevertheless, the greatest increase in survival was observed when both GSTs and HPV18 E7 were present (Figure 4F).

### Loss of GSTs inhibits proliferation and survival of cervical cancer cell lines

**Figure 5:**
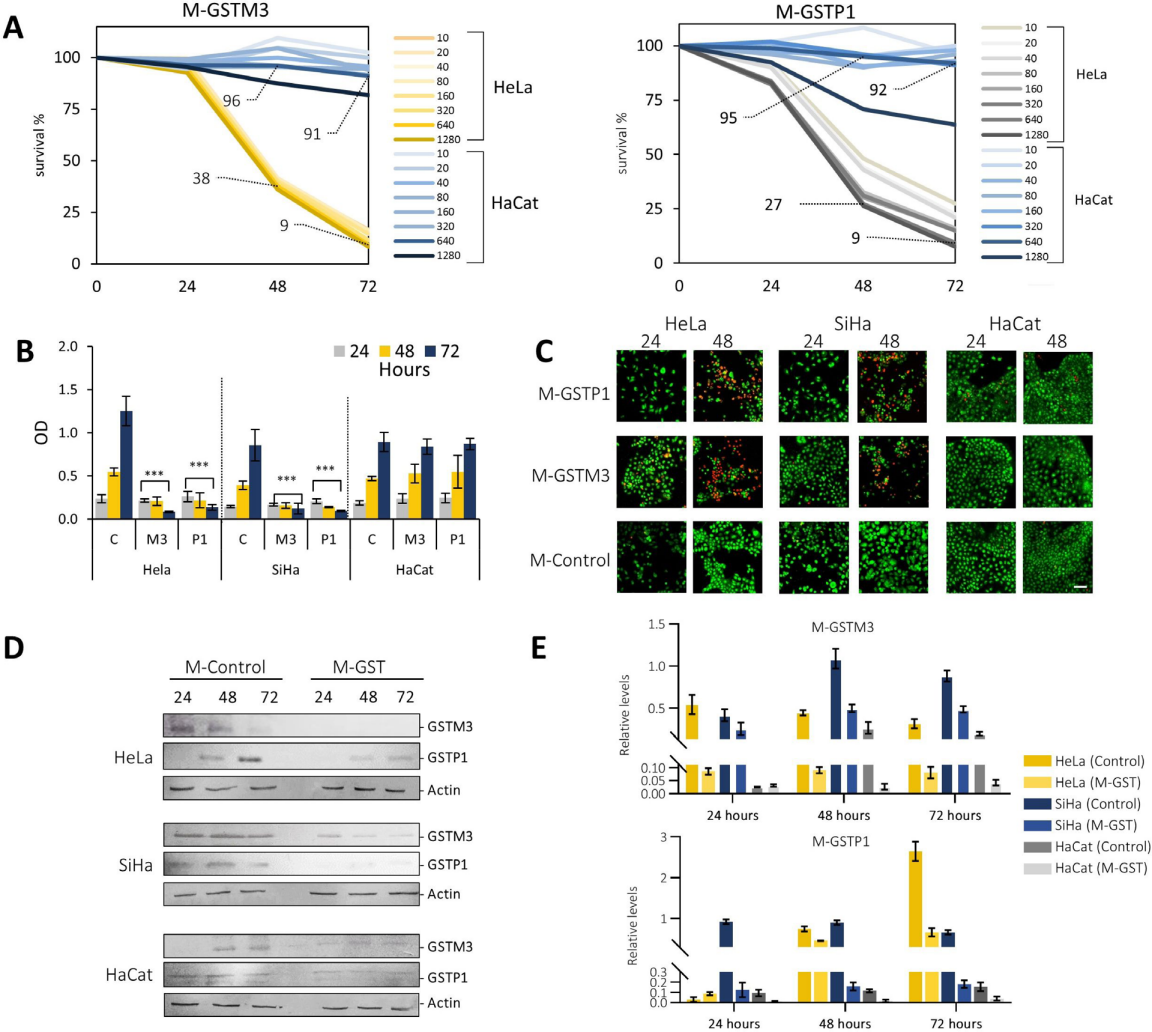
Knock-down of GSTM3 and GSTP1 affect the viability of cervical cancer cell lines in culture (**A**) Gene knock-down experiments of GSTM3 and GSTP1 in HeLa (yellow tone lines) and HaCaT cell lines (blue tone lines, used as a control), using a 10–1280 ng/mL gradient concentration. No changes were observed in cell proliferation 24 hours after treatment. At 48 hours and 640 ng/mL, minor changes in HaCaT cells (survival of 96% in GSTM3 and 95% in GSTP1), and at 72 hours and the same concentration, survival of 91% in GSTM3 and 94% in GSTP1. A major decrease in HeLa cells (survival of 38–9%, in GSTM3, and 27–9% in GSTP1, at 48 and 72 hours respectively) was observed. (**B**) Viability assay treated with M-Control or M-GSTs (at 640 ng/mL), determined by crystal violet staining. (**C**) Live/dead assays were determined by SYTO 9 staining in cells treated with M-GSTs control or M-GSTs (640 ng/mL), scale bar 50 µm. All assays were performed in triplicate. (**D**–**E**) Inhibition of GSTs with morpholino treatment in CC cell lines Relative protein expression. Western blot of morpholino assay inhibition of GSTs in CC cell lines (HaCaT used as negative control of CC). Cell lysates were subjected to western blot with 20 µg of protein per sample. The data are presented as means (SD) of three independent experiments *P*-value < 0.05 was considered statistically (^***^P-value < 0.001 with respect to control).

GSTs play important roles in the regulation of MAP kinases and NF-κB pathways, activating cellular maintenance, proliferation and apoptosis evasion [20]. In order to evaluate the effect of GSTM3 and GSTP1 in CC cell lines, we inhibited the expression of both proteins by means of morpholino antisense oligonucleotides. Three morpholinos were designed, two to specifically knockdown the proteins and one with a random sequence used as a control (M-GSTM3, M-GSTP1, and M-Control). We first evaluated eight doses for each morpholino in two cell lines, HeLa and HaCaT. The doses used ranged from 10 to 1,280 ng/mL incorporated into the culture media, and we evaluated cell proliferation at three different times, 24, 48 and 72 hours. We observed that HaCaT cells were not affected by treatment with M-GSTM3 during the period of analysis. We only noticed a slight loss of survival at the highest dose (1,280 ng/mL). In HeLa cells, we noticed losses of viability after 48 hours of treatment at all doses of M-GSTM3 (Figure 5A). After 72 hours, the highest doses of treatment (640 and 1,280 ng/mL) showed a survival less than 10% compared to control cells. Similar results were obtained for M-GSTP1 treatment in both cells. In order to evaluate the cellular response in other cell lines, we selected the dose of 640 ng/mL, because that was the highest dose that did not affect the unresponsive HaCaT cell line. Additionally, we treated to the cervical cancer cell line SiHa with 640 ng/mL (Figure 5B). We observed highly similar response between cancer cell lines, indicating that both GSTs proteins are essential for cellular survival in cervical cancer cells, but not for the non-cancerous HaCaT cells. To validate the effectiveness of the knock-down treatment, we performed western blot analysis in the three cell lines for both proteins (Figure 5D–5E). The immunoblotting revealed that both proteins were indeed down-regulated during all times of the treatment in the three cell lines. Additionally, we evaluated cell viability in the three cell lines after 24 and 48 hours of treatment at the dose of 640 ng/mL of the two morpholinos (Figure 5C). We carried out a live/dead assay based on Syto9/Propidium iodide staining. The results confirmed that HaCaT cells were not affected by the treatment. Both cancer cells lines were affected in a similar way. Altogether, these results suggest that HaCaT cells possess an alternative cell maintenance mechanism that is compromised in cervical cancer cells.

### Loss of GSTs inhibits tumor progression in cervical cancer

**Figure 6:**
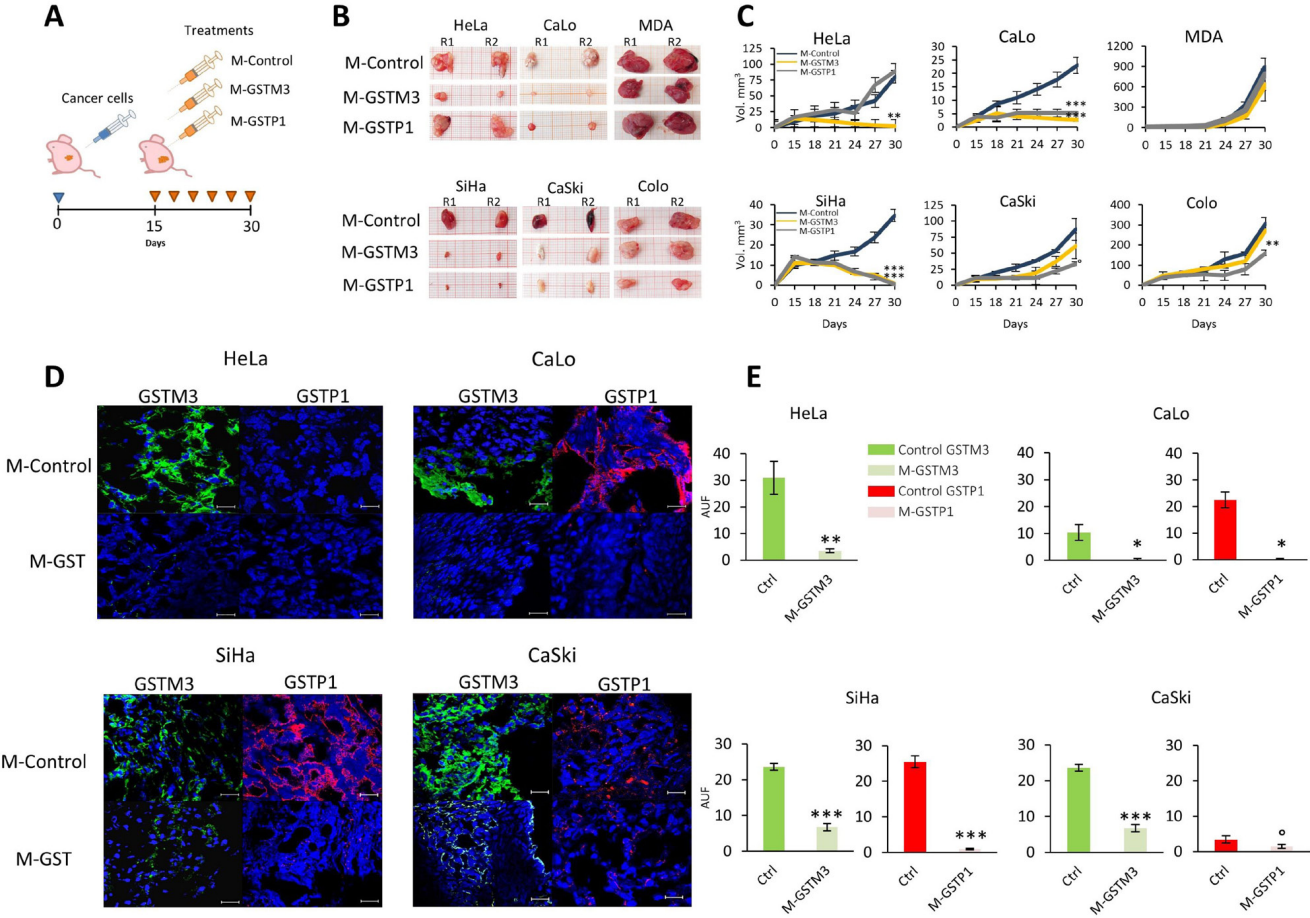
Knock-down of GSTM3 and GSTP1 affect TP in CC tumors (**A**) Nu/Nu mice were injected with 10^7^ tumor cells from the four CC cell lines, HeLa, CaLo, SiHa and CaSki and two non-CC lines (MDA and COLO). After 15 days of growth, GSTP1 and GSTM3 were knocked down in tumors (every third day) using different morpholinos. Morpholino treatment consisted on delivery via a vehicle containing PBS/morpholino 400 ng/dose in the final dose of 50 µL, next to localized tissue. In the day 30, all tumors were collected for further analysis. (**B, C**) Knockdown effect over TP, showed impaired tumor xenograft growth in CC cell lines positives to GSTs with treatments of morpholino M-GSTM3, M-GSTP1. (**D, E**) Protein expressions was confirmed by IHC of GSTs. Assays were performed in duplicate. Scale bar 20 µm. The data are presented as means (SD) of two independent experiments (°*p* < 0.05; ^*^p < 0.025; ^**^p < 0.01; ^***^p < 0.001).

To investigate the role of GSTs during TP, we next examined the effects of morpholino treatments in a murine model (Figure 6A). To this end, we used morpholino antisense oligonucleotides (M-GSTM3, M-GSTP1, and M-Control) to treat four CC cell lines (two HPV16-positive lines, SiHa and CaSki, and two HPV18-positive lines, HeLa and CaLo), as well as two negative control of CC cell lines, breast (MDA-MB-231), and colon (COLO 205) cancer tumors. The results of our *in vivo* and *in vitro* analyses were correlated with each other, showing a drastic decrease in volume CC tumor cell lines (Figure 6B–6C). However, the results for HeLa tumors were different from those performed *in vitro*. HeLa tumors express only GSTM3 and not GSTP1 (Figure 5D–5E). M-GSTP1 treatment of HeLa tumors did not affect TP, confirming that GSTP1 is not expressed in these tumors. Treatment with M-GSTM3 in HeLa tumors drastically decreased the tumor volume. Compared to treatment with the random sequence morpholino, the volume of M-GSTM3 treated HeLa tumors was 14-fold lower (Figure 6B–6E).

In CaLo tumors, we found that both GSTM3 and GSTP1 were expressed (Figure 6D–6E). The treatment of these tumors with morpholinos against GSTM3 and GSTP1 resulted in a decrease of tumor volume by 10- and 6-fold, respectively (Figure 6B–6C). In the case of SiHa tumors, which express both proteins (Figure 6D–6E), we observed the greatest decreases in tumor volume after treatment with both morpholinos, with decreases of 43- and 62-fold for M-GSTM3 and M-GSTP1, respectively (Figure 6B–6C). CaSki also expresses both proteins in control tumors. In treated tumors, we observed that the levels of GSTM3 and GSTP1 did not decrease as much as in other tumors that express these proteins (Figure 6D–6E). The treatment with M-GSTP-1 resulted in a reduction of the tumor volume by 2.6-fold compared to the control. In the case of M-GSTM3 treatment, we were unable to observe significant differences between control and M-GSTM3-treated tumors in our experimental conditions (Figure 6B–6C). Probably, the low efficiency of protein reduction the treatment, particularly for GSTM3, is responsible for the low response in the tumor reduction. In agreement with this observation, we hypothesize that the remaining GSTM3 is sufficient to provide at protective effect to tumor cells.

In addition, we explored the response to treatment of tumors from two cell lines of different origins, MDA-MB-231 from breast cancer and COLO from colon cancer. Both tumors exhibit low expression of GSTP1 compared to CC tumors (Figure 6D–6E). However, treated COLO tumors had 1.9-fold lower levels than control (Figure 6B-C). In the case of MDA-MB-231, the levels of GSTP1 were barely detectable in control tumors, and as a consequence, the treatment with M-GSTP1 did not affect normal TP (Figure 6B–6C). Tumors from both cell lines expressed GSTM3 and in both cases, the treatment with the morpholino significantly reduced the levels of the protein. However, we were unable to correlate this down-regulation with TP.

### GSTM3 and GSTP1 regulate the MAP kinase proteins pJNK and pp38

**Figure 7:**
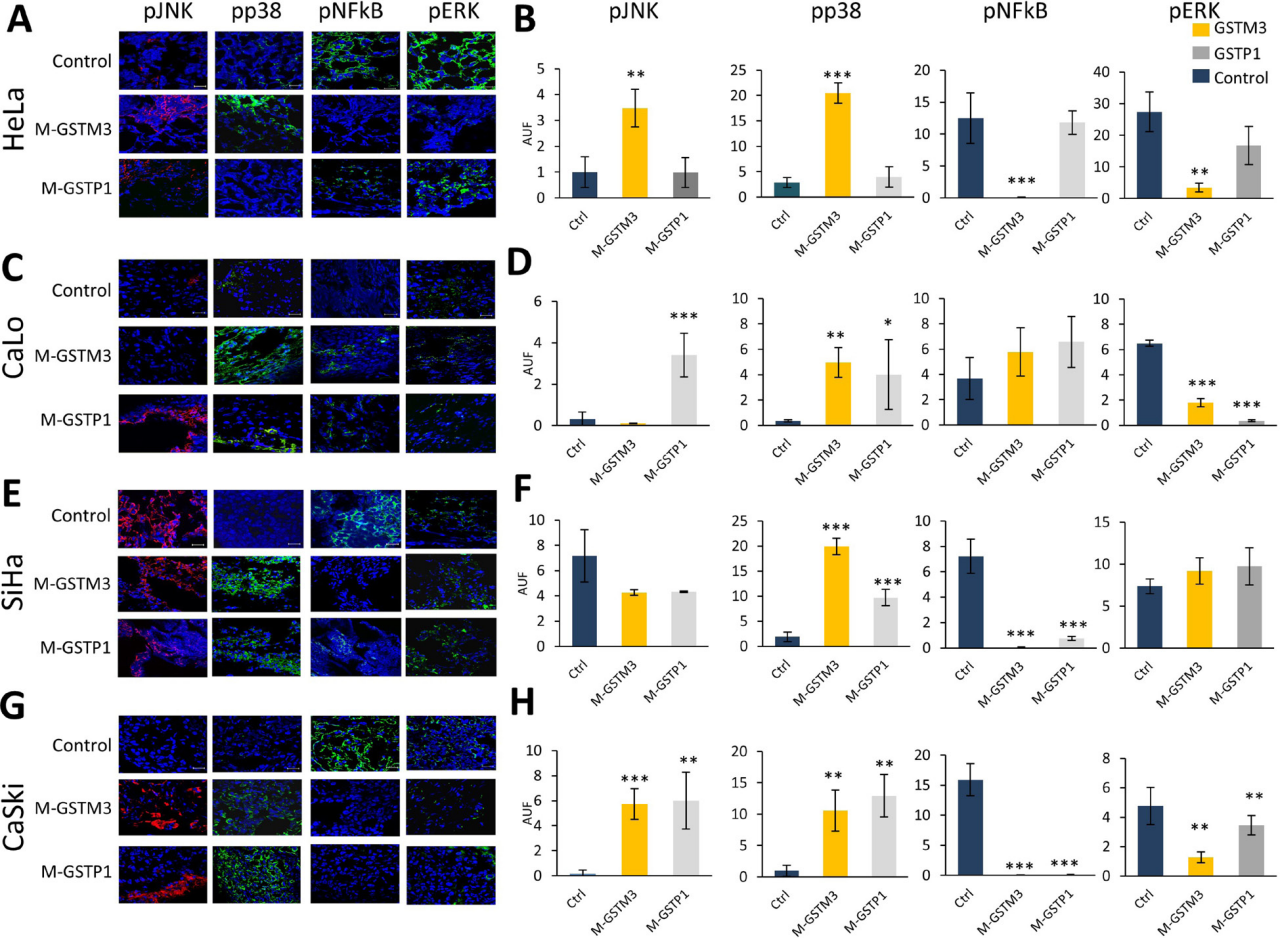
Knock-down of GSTM3 and GSTP1 affect the activation of pJNK, pp38, pNF-κB, pERK in CC tumors (**A–B**) HeLa tumors knockdown with effect on pJNK, pp38, pNF-κB and pERK. (**C–D**) CaLo tumors knockdown with effect on pJNK with M-GSTP1, pp38 and pERK in both treatment. (**E–F**) SiHa tumors knockdown with effect on pp38 and pNF-κB in in both treatment. (**G–H**) CaSki tumors knockdown pJNK, pp38, pNF-κB and pERK. Assays were performed in duplicate. Scale bar 20 µm. The data are presented as Means (SD) of two independent experiments (^*^p < 0.01, ^**^p < 0.005, ^***^p < 0.001).

Previous research has demonstrated that GSTP1 protein expression can affect MAP kinases, leading to decreases in pJNK and pp38 phosphorylation and activation [25, 36, 48, 49]. Hence, we sought to examine the effects of GSTM3 and GSTP1 knockdown on pJNK and pp38 activation and the phosphorylation of p65 and pERK (from the NF-κB pathway) during CC TP (Figure 7). To this end, we analyzed the protein expression through immunohistochemical assays in all CC tumors treated with morpholinos (M-GSTM3, M-GSTP1, and M-control). M-GST treatments resulted in the phosphorylation and activation of pJNK and pp38 MAP Kinases. HeLa tumors only expressed GSTM3 and therefore only responded to treatment with the M-GSTM3 morpholino, showing increased phosphorylation of JNK and p38 (Figure 7A–7B). Both tumors from CaLo and SiHa, only showed phosphorylation of p38 alone, following both treatments with M-GSTM3 and M-GSTP1, CaLo (Figure 7C–7D); and SiHa (Figure 7E–7F). For CaSki tumors, both MAPKs were up-regulated upon treatment with M-GST (Figure 7G–7H).

### GSTM3 and GSTP1 regulate cell survival by inactivating NF-κB and pERK

To investigate the role of GTSs in programmed cell death and cell survival, we also examined the inactivation of ERK or NF-κB protein p65 (Figure 7). HeLa tumors treated with M-GSTM3 showed inactivation of both proteins (Figure 7A–7B). Only pERK was inactivated in CaLo tumors following treatment with either of the GST oligonucleotides (Figure 7C–7D). For SiHa tumors only NF-κB was inactivated by either of the treatments (Figure 7E–7F). In CaSki tumors, both proteins became inactivated following either treatment (Figure 7G–7H). We suggest that inhibition of GSTM3 and GSTP1 proteins induced apoptosis and decreased cell survival via the NFκB and MAP-kinase pathways.

### GST expression analysis in CC patient tissue specimens

**Figure 8:**
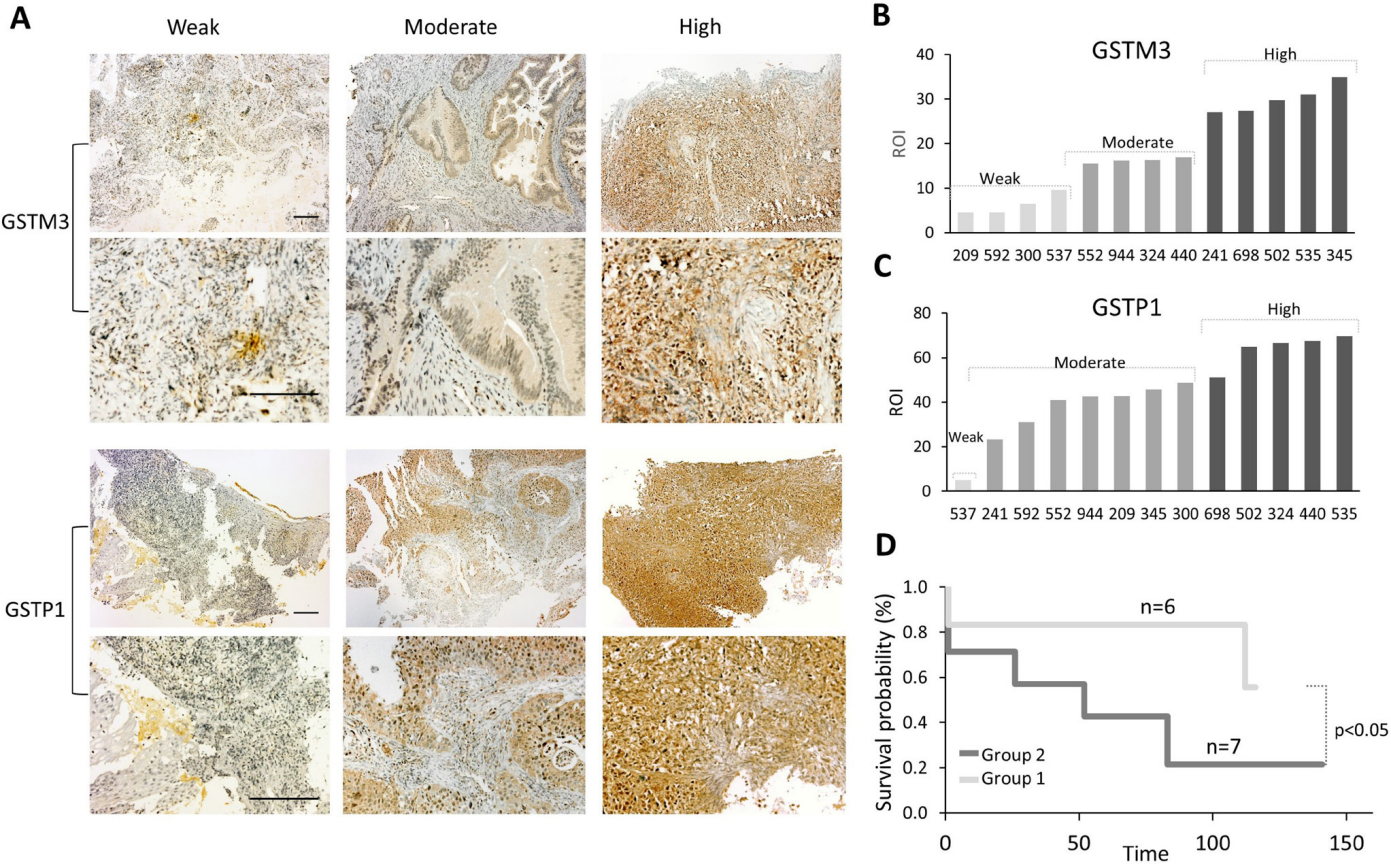
Correlation between expression of GSTs proteins and survival of CC patients (**A**) ROI of GSTM3 and GSTP1 in 13 CC patients. ROI Percentage of GSTM3 was classified as weak (≤10%), moderate (10–20%), and high (21–50%). (**B**) ROI Percentage of GSTP1 was classified as weak (≤10%), moderate (20–50%) and high (51–100%). (**C**) Representative specimens of invasive CC with different GST expressions (weak, moderate and high). Scale bar 100 µm. (**D**) Kaplan–Meier survival plot, for the advanced stage of cervical cancer according to the protein expression levels of GSTM3 and GSTP1 (log-rank test, *p* < 0.05). Group 1 (WM-M): weak-moderate ROI of GSTM3 and moderate of GSTP1; and group 2 (MH-H): moderate-high ROI of GSTM3 and high ROI of GSP1.

It is known that the expression of GSTs in some types of cancer is considered to be a sign of a poor prognosis, [16–19] and that these proteins can be responsible for the chemoresistance observed in many CC patients. To understand the role of GST protein expression in chemoresistance in patients with CC, we performed a follow-up study of 13 patients suffering from CC who had undergone chemotherapy (Supplementary Figure 5A–5B). Protein expression analyses were performed for GSTM3 and GSTP1 using immunohistochemistry (IHC). In this study, we analyzed the percentage of the region of interest (ROI) that was immunopositive. Remarkably, all patients expressed both proteins, but with great variability regarding the percentage of the ROI (Figure 8A, Supplementary Figure 5A–5B). We categorized the patients into three arbitrary groups based on the percentage of involvement: weak, moderate and high for GSTM3 and GSTP1 (Figure 8B–8C). Then, we performed an association analysis of GST expression and patient survival and generated two groups: weak-moderate for GSTM3 and moderate for GSTP1 (WM-M), and another group with moderate-high values for GSTM3 and high values for GSTP1 (MH-H) (Figure 8D). The results showed that the expression of GSTM3 and GSTP1 might significantly influence the survival of patients with CC. There was a clear correlation between patient survival and the expression of GST proteins, with the patients who exhibited weak-to-moderate (WM-M) expression showing a significantly higher survival rate than patients exhibiting moderate-to-high (MH-H) GST expression (Figure 8D, Supplementary Table 12).

## DISCUSSION

In this study, we developed a tumor progression model by using two cervical cancer cell lines (SiHa and HeLa) xenotransplanted in athymic nude mice. To analyze the TP proteome, we performed a 2D-PAGE analysis comparing three different ages of tumors from the two CC cell lines. We observed that two members of the GST (GSTM3 and GSTP1) family had increased expression throughout the time period. HeLa tumors expressed GSTM3, and SiHa tumors expressed both GSTs. Previous studies indicated that GSTP1 plays a regulatory role through its interaction with TRAF2, affecting apoptotic signal activation [25, 26]. Based on these findings, we wondered if GSTM3 plays a similar role to GSTP1. For this purpose, we performed a bioinformatic analysis using a protein-protein interaction network. The analysis suggested that GSTM3 interacts with TRAF6, and we confirmed this interaction by means of a co-IP assay involving GSTM3 and TRAF6 proteins in HeLa tumors.

On the other hand, it has been reported that TRAF6 stimulates the JNK pathway [25, 50], and is selectively required the for induced activation of p38 via the TRAF6-ASK1 axis [51]. TRAF6 and TRAF2, in turn, recruit the TAK1 and IKK complexes, leading to the activation of NF-κB [52]. TRAF2 and TRAF6 have also been demonstrated to bind and activate ASK1 [51, 53]. In consequence, if GST proteins are interacting with TRAF2/6, they would be regulating JNK, p38, and NF-κB during TP. Therefore, we analyzed the proteins related to NF-κB, JNK, and p38 in all three stages of TP. The results showed that during TP of CC tumors, GST could be interacting with TRAF2 and TRAF6 and hence, could regulate cell survival, cell proliferation and apoptosis evasion (Figure 3B). These results were consistent with those of a previous study, which reported that the interaction of TRAF2 and GSTP1 can block apoptosis and stimulate cell survival [25]. In a similar, way our results suggest that the interaction of GSTM3 and TRAF6 plays an anti-apoptotic role based on interactions with c-Jun N-terminal kinase (JNK) [26].

On the other hand, it is known that the activation of the TLR4 pathway is not only driven by the presence of lipopolysaccharide (LPS) from bacterial infections [44] but also by endogenous activators as HSP60 and HSP70 [45, 46]. Here we performed an identification analysis of secreted proteins from both SiHa and HeLa CC cell lines, in culture and in tumors. In both experiments, we were able to identify HSP60 and HSP70 as part of the secretome in both cells. These results indicate that the secretion of HSP60 and HSP70 could potentially activate TLR4 signaling and therefore induce cell survival and apoptosis evasion during TP.

Previous reports indicated that the interaction of GSTP1 with the viral oncoprotein E7 from HPV16 provides the cancer cells with a better adaptation capacity under stress [36]. Here we demonstrated that GSTM3 interacts with E7 from HPV18. In order to clarify whether this interaction can provide cells with an advantage in terms of cell survival under stressful conditions, we performed survival experiments in the presence of GSTM3, GSTP1, and E7 from HPV18. The results indicated that incorporation of any of these proteins provided a survival advantage and the incorporation of all three proteins slightly increased the survival, compared to individual proteins, in cells treated with UV and cisplatin. Considering these findings, we concluded that even when GSTs and E7 oncoproteins from HPVs interact, the presence of each of these proteins individually increases the survival of the cells, indicating that the interaction is not crucial in the response to stressful conditions.

In many CC patients with observed drug and chemotherapy resistance, the expression of GSTs proteins contributes to cancer cells survival and can be responsible for this observation [18, 19]. In addition, GSTs expression in some types of cancer is already considered to be a sign of a poor prognosis [16, 17]. Considering this, we inhibited the expression of GSTM3 or GSTP1 in both cell cultures and xenotransplanted tumors. In culture, cancerous SiHa and HeLa cells were drastically affected by the knock-down of both GSTs, while the non-cancerous HaCaT cells were not affected by the inhibition of these proteins. These findings point to GSTM3 and GSTP1 as being crucial for the survival and proliferation of cancerous cells. In xenotransplanted tumors, we observed that in those from cervical cancer cell lines that expressed at least one of these proteins, the tumor volume was drastically decreased after treatment with morpholinos.

**Figure 9:**
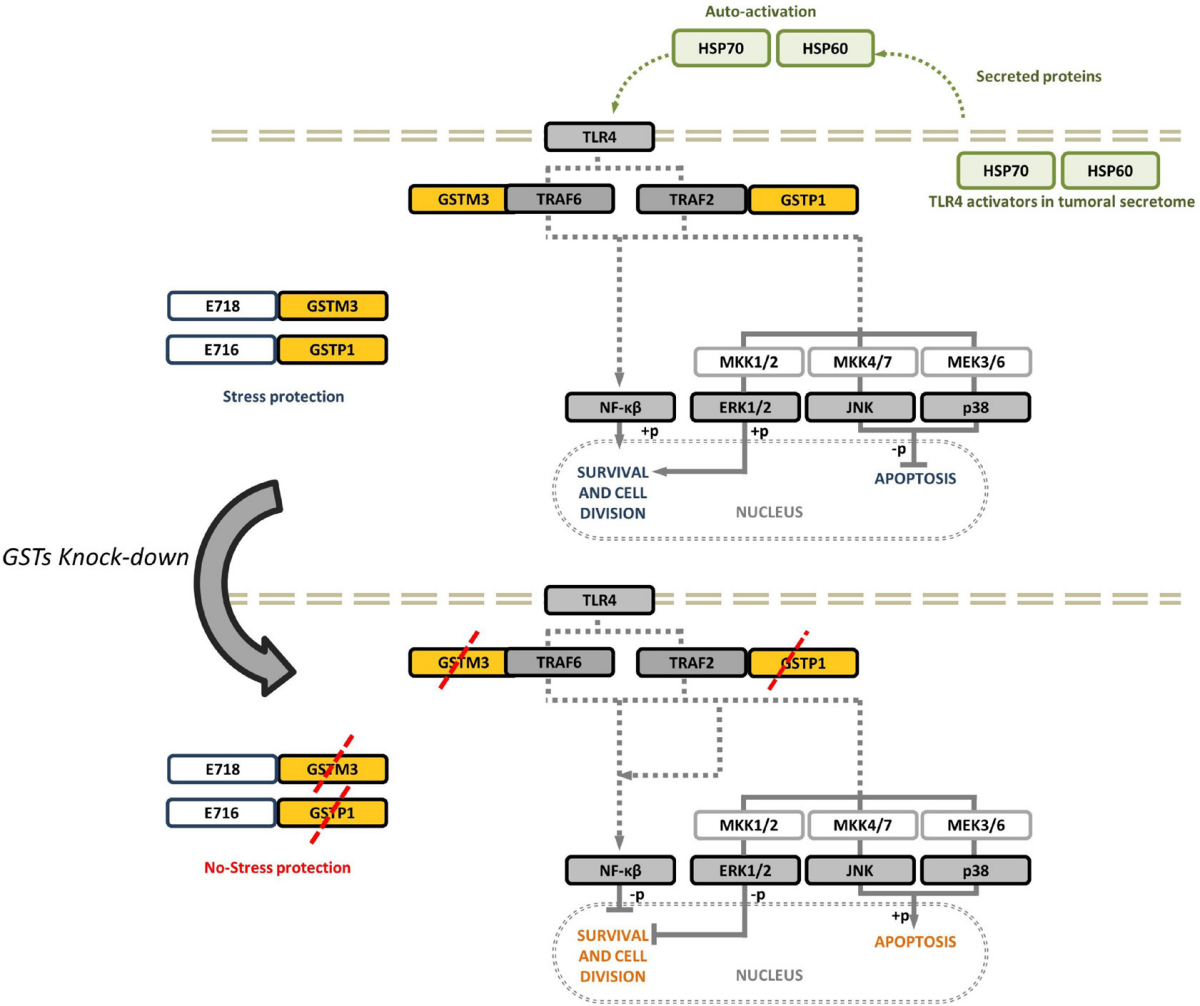
Schematic representation of the role of GSTs proteins in CC tumors During the progression of cervical cancer, several processes such as cell survival, proliferation, and apoptosis evasion via MAPK and NF-kB are stimulated by the presence of GSTM3 and/or GSTP1. Knock-down of GSTM3 and GSTP1 affect the activation of MAPK and activated apoptosis through activation of MAPK or phosphorylated inhibition of NF-kB and ERK.

We suggest that GSTs expression (GSTM3 or GSTP1) could be involved in modulating detoxification processes in cancer cells, and therefore, may participate in the survival response to conventional chemotherapy in patients. GSTs have been proven to regulate kinase signaling pathways, and it has been shown that GSTM3 and GSTP1 inhibit JNK signaling and prevent transcriptional activation of downstream cell stress pathways [20]. These proteins might co-evolve during tumorigenesis to allow the cells to adapt to stressful conditions associated with the tumor microenvironment. This adaptation mechanism would further allow the cancer cells to respond to xenobiotic agents, such as chemotherapy [48, 49, 54]. Here we demonstrated that the inhibition of GSTM3 or GSTP1 activates JNK and p38 signaling leading cells to apoptosis and therefore decreasing the tumor volume. On the other hand, we observed that inactivation of NF-κB and/or ERK following inhibition of GSTs inhibited cell survival. We analyzed tumors from MDA-MB-231cells a triple negative breast cancer (TNBC), and we did not observe GSTP1 protein expression during TP or changes in tumor growth after treatment with anti-GSTP1 morpholine under our experimental conditions. Here we proposed a mechanism by means of which the cervical cancer cells use GSTs to avoid apoptosis and to activate cell survival and proliferation. In addition, this response is affected by the knock-down of these proteins (Figure 9). These findings illustrate the crucial roles of at least these two GST proteins in the progression of cervical cancer and its resistance to stressful conditions.

It has been demonstrated that GSTM3 is associated with cancer risk at a genetic level, where the association of certain polymorphisms and/or mutations increases the risk of different types of cancer, such as lung cancer [23], prostate cancer [21], and colorectal cancer [17, 24]. The protein expression of GSTM3 has been analyzed in colon cancer, and its overexpression is considered a marker of regional lymph node metastasis [17]. On the other hand, subexpression of GSTM3 is associated with better survival in urinary bladder cancer [27]. Genetic studies of CC have demonstrated that polymorphisms in the GSTM3 gene are associated with a greater risk of developing this cancer [55]. However, there have not been sufficient studies addressing the correlations with clinical endpoints. In the present study, we showed that there is a strong association of GSTM3 and GSTP1 protein expression and survival of patients. Our results agree with clinical data, in which the survival of CC patients was associated with high levels of GST protein (Figure 7C). These data were also in agreement with studies on bladder [27] and colon cancer [17] in which overexpression of the GSTM3 protein was found to be associated with a reduced survival rate of patients. Accordingly, here we propose that both GSTM3 and GSTP1 can be added to the list of novel and promising therapeutic targets and/or prognostic factors for CC patients.

## MATERIALS AND METHODS

All animal experiments were performed with approval from the institutional research bioethics committee of the Instituto de Biotecnología at Universidad Nacional Autónoma de México (UNAM), Cuernavaca, Morelos and the Instituto Nacional de Ciencias Médicas y Nutrición Salvador Zubirán (INCMSZ). The institutional review board approved all studies involving human tissues for human subject research of the Instituto Nacional de Cancerología, Mexico City (INCan). Informed consent was obtained from all patients before the biopsy.

### Cell culture

Cervical cancer cell lines (SiHa and CaSki, HPV16-positive, and HeLa and CaLo, HPV18-positive) were provided by Dr. Jorge Flavio Rincon and M.S. Rosalva Rangel Corona at the Laboratorio de Oncología Molecular, Unidad de Diferenciación Celular y Cáncer, FES-Zaragoza, UNAM. Dr. Alejandro Zentella Dehesa at the Unidad de Bioquímica del Instituto Nacional de Ciencias Médicas y de Nutrición Salvador Zubirán, provided the MDA-MB-231(breast cancer) and COLO 205 (colon cancer) cell lines. The HaCaT cell line was provided by Dr. Alejandro García Carrancá at the Instituto Nacional de Cancerología, Mexico City, México. All cells were tested for *Mycoplasma sp.* by PCR and then maintained in FBS-free advanced RPMI 1640 medium (Gibco, Invitrogen) and supplemented with 2 mM L-glutamine and 1% v/v antibiotic-antimycotic (Gibco, Invitrogen) and maintained at 37° C in a humidified incubator with 5% CO_2_.

### Mice tumor xenografts

4-6-week-old female athymic nude mice (BALB/c nu/nu) were injected subcutaneously with 10^7^ tumor cells in 500 μL FBS-free RPMI 1640 medium and collected in 30, 45 and 50 days. Tumors were measured using a Vernier caliper, calculating ellipsoid [28] volumes as where L: long, W: wide, and H: high.

### Tumor protein extraction, proteomic analysis, and mass spectrometry

Tumor samples were macerated in liquid nitrogen, and a protease inhibitor cocktail (Complete tablets, Roche) followed by sonication on ice 3-5 times for 5 seconds. We subsequently conducted phenolic protein extraction [29]. The protocols followed for sample preparation, preparative two-dimensional gel electrophoresis (2D-PAGE), image analysis and protein identification through MALDI mass spectrometry have been previously reported [30, 31] (more details see: MIAPE ID: 821 2D-PAGE). Briefly, for 2D-PAGE 500 μg of total proteins were isoelectric focused along a linear pH range of 3–10 and the second dimension was performed in a 12% acrylamide SDS-PAGE. Digital images of the 2D-PAGE were acquired using a GS-800 Calibrated Densitometer (BioRad). Three gels obtained from three different assays were analyzed, and image comparison was performed with PDQuest software (BioRad) (more details see MIAPE ID: 10290, MALDI-TOF; MIAPE ID: 9905 MASCOT).

### *In vitro* and *ex vivo* extracellular protein extraction

Cell lines were cultured with advanced RPMI 1640 serum-free until 70–80% of confluence. The medium was removed and were rinsed three times with saline solution sterile: NaCl 0.9% (w/v). After washings, FBS-free RPMI 1640 medium without phenol red fresh (Gibco), was added and incubated for 20 h. Later the medium was recovered and centrifuged at 2,000 g for 5 min. The supernatant was passed through a 0.22 μm pore-size membrane PVDF (Millex, Millipore) and stored –70° C until further use.

For extracellular proteins from xenograft tumors, HeLa and SiHa tumors were inoculated with 10^7^ cells. After 30, 45 and 50 days post-inoculation tumors were collected and washed 3 times with saline solution. The followed procedure to extract secreted proteins from tumors was performed as previously described for the cells in culture and the supernatant was stored –70° C until further use.

### Identification of secreted proteins via LC-MS/MS

Secreted proteins from cell lines separated in SDS-PAGE and stained with Coomassie brilliant blue stain (more details see: Supplementary Figure 3). Each lane containing 30 μg of proteins was cut into 20 slices and digested with trypsin. Generated peptides were analyzed in a nanoLC-MS/MS system (Q-TOF Synapt G2 MS; Waters). The peptide and protein identification was performed using MASCOT search engine through the MASCOT Distiller interface (Matrix Science). The queried database was the Swiss-Prot. The mass spectrometry proteomics data have been deposited to the ProteomeXchange Consortium via the PRIDE [32] partner repository with the dataset identifier PXD005466.

### Western blot

The following commercial antibodies were used: anti-GSTM3 (Abcam, ab67530, 1:10,000), anti-GSTP1 (Abcam, ab53943, 1:10,000), anti-TLR4 (Biolegen, 312804, 1:10,000), anti-TRAF6 (Abcam, ab13853, 1:10,000), anti NF-kB p65 (sc-378,1:1,000), anti IKB-α (sc-371, 1:1,000), anti-JNK (sc-1648, 1:1,000), anti-ERK (sc-94, 1:1,000), anti-p38 (sc-535, 1:1,000), anti-NF-κB phospho p65 (sc-101752, 1:1,000), anti-phospho-JNK (sc-6254, 1:1,000), anti-phospho-ERK (sc-7383, 1:1,000), phosho-p38 (sc-7973, 1:1,000), anti-phospho-IKB-α (Cell signaling, 1;1000), anti-HSP70 and HSP60 (Biolegen, 648005 and 681502, 1:10,000), HPV18 E7 (Abcam, ab38743, 1:1,000), anti-His tag antibody (Invitrogen, 372900, 1:5,000). Cells were lysed in a buffer containing 100 mM Tris pH 8.6, 4% SDS, 100 mM DTT, protease inhibitor cocktail (Complete tablet, Roche) and 20 sonication pulses for 1 second for DNA fragmentation. Proteins were resolved by electrophoresis on 12% or 15% SDS-PAGE and transferred to nitrocellulose membranes using semi-dry system. Blots were blocked with 5% non-fat milk in Tris-buffered saline containing Tween 20 (TBST) for 15 min at 4° C, washed tree times in TBST, and probed with primary antibody diluted and incubated at 4° C overnight, membranes were incubated with peroxidase-conjugated secondary antibody for 2 h and then membranes were incubated with Carbazole solution (27.2% Stock Carbazole, 72.6% Acetate buffer, 0.2% H_2_O_2_), Carbazole Stock: N, N-Dimethylformamide ≥98% and 3-Amino-9-ethylcarbazole (Sigma-Aldrich) 1:8 (w/v) for generate colored stain. Relative quantifications were performed using ImageJ software.

### Co-immunoprecipitation and immunoblotting

HeLa tumor was collected at 50-day and stored at 80° C until use. Following the tumor sample was macerated in liquid nitrogen, and lysed with 500 μL of RIPA buffer (10 mM Tris, 1 mM EDTA, 1% NP40, 0.1% Sodium deoxycholate, 140 mM NaCl) and supplemented with protease and phosphatase inhibitors (10 mM β-glycerolphosphate, 10 mM Na_3_VO_4_, 10 mM Sodium Fluoride). The total cell lysates were centrifuged at 13,000 g for 5 min to pellet the insoluble material. The lysates were precleared 2-hour incubation with protein A sepharose and normalized for total protein concentration (10 μg of protein) using SDS-PAGE. The proteins candidate antibodies (GSTM3 and TRAF6) were immunoprecipitated by incubating lysates with 6 μl of antibody-conjugated sepharose overnight at 4° C. The beads were washed 3 times with 500 µL of lysis buffer. Co-immunoprecipitating proteins were resolved on 12% SDS-PAGE. Levels of GSTM3 and TRAF6 were detected by immunoblotting using anti-antibodies previously described above.

### Bioinformatics analysis and structural superposition

A gene ontology (GO) analysis of the 39 common proteins identified was performed using the GeneCodis website [33]. A protein-protein interaction network model using Cytoscape 2.5.1 (www.cytoscape.org) and bisogenet plug-in was employed to obtain the protein-protein interactions information about GSTM3 and GSTP1 [34]. The structural superposition of GSTP1 and GSTM3 was performed with MAMMOTH (https://ub.cbm.uam.es/software/online/mamothmult.php) and was validated and visualized with Swiss PDB viewer software (DeepView) v4.1 [35]. We obtained the GSTP1 model docked with the HPV16 E7 CR3 dimer model from the Mileo group [36], and the GSTM3 model was obtained from the PDB database. We generated a structural alignment to improve the fit between GSTP1 (known structure) and GSTM3 and between HPV16 E7 (known structure) and HPV 18 E7. The server built the models based on the provided alignments [35].

### UV assay

Radiation was delivered from UV-lamps at 100% power (450 µW/cm^2^, at 27 mm) according to the supplier’s specifications (InGeniuous LHR equipment; Syngene). The MDA-MB-231 cells were exposed to radiation over the surface of a transilluminator for 0, 10, 15, 30 or 60 seconds. A total of 10^4^ cells were cultured in 24-well plates (Nunclon). For the subsequent experiments and treatments, the cells were exposed to UV light for 15 seconds.

### Cisplatin assay

Cell lines were cultured in FBS-free advance RPMI 1640 (Sigma) medium at 37° C in a humidified 5% CO_2_ atmosphere. Drug sensitivity was evaluated at the 6 mM concentration of cisplatin according to previous studies [37]. To measure cell viability, a culture was initiated at 10^4^ cells/mL and was analyzed every day until the eighth day. The number of cells was determined by crystal violet staining [38]. The O.D. was measured at 550 nm using a microplate reader (Bioteck).

### Phenotypic analysis through exogenous complementation of recombinant proteins

Stress sensitivity (UV radiation and drug treatments) was evaluated through exogenous complementation with human recombinant protein and the Lipofectamine 3000 transfection reagent (Invitrogen). A 10 µL aliquot of the Lipofectamine 3000 and P3000 reagents (Invitrogen) was mixed with 1 µg of human recombinant GSTM3 (Abcam), GSTP1 (Abcam), HPV18 E7 (ProteinX) or a mixture of these proteins in 250 µL of serum-free advanced RPMI 1640 medium, which was then incubated for 5 min at room temperature and added to 10^4^ MDA-MB-231 cells cultured in 24-well plates (Nunclon), followed by another 5 min incubation [39]. After incubation with the Lipofectamine/recombinant protein mixture, the cells were exposed to UV radiation or drug treatments under FBS-free conditions. Cell viability was analyzed until the end of the recovery period (24 hours after UV, four days after cisplatin). The cell number was determined by crystal violet staining [38]. The O.D. was measured at 550 nm, using a microplate reader (Bioteck).

### Yeast plasmid construction, transformation, and recombinant protein expression

A gene expression of GSTM3 was inserted into the pYES2 plasmid in *S. cerevisiae*, and additional histidine (N-6x his) codons were introduced (more details, see Supplementary Table 10 and Supplementary Figure 3). The total protein was obtained through cell lysis using Lyticase as described by the supplier (Sigma-Aldrich), and maceration with liquid nitrogen. The cell lysates were centrifuged at 5,520 g for 5 min, and the supernatant was stored at –20° C. The recombinant protein was purified by metal chelate chromatography using nickel beads as described by the supplier (PureProteome, Merck Millipore). Eluted proteins were visualized in 12% SDS-PAGE gels through Coomassie brilliant blue staining. The band corresponding to GSTM3 was confirmed by mass spectrometry (MALDI-TOF, Bruker Daltonics).

### Human cell line transformation, protein purification, and pull-down

The gene sequence of HPV18 E7 was obtained using the pBR322HPV18 plasmid (ATCC, 45152D) and transfected with CMV-pcDNA3.1 plasmid using Lipofectamine 3000 (Life Technologies) in HeLa cells. The 18 to 26 nt oligos were designed with SnapGene Viewer 2.2.2 (GSL Biotech LLC), with the addition of *Hind*III and *Bam*HI sites and C-6x His-tag (Supplementary Table 11). The recombinant protein HPV18 E7 was purified by metal chelate nickel beads as described by the supplier (PureProteome, Merck Millipore). Eluted proteins were visualized in 12 and 15% SDS–PAGE gels through Coomassie brilliant blue staining. The band corresponding to HPV18 E7 was confirmed by western blotting using HPV18 E7 (Abcam, ab38743, 1: 1,000) and an Anti-His tag antibody (Invitrogen, 372900, 1: 5,000).

### *In vitro* and *in vivo* knockdown using morpholino oligonucleotides

Morpholino constructs were designed to target human GSTM3 and GSTP1 transcripts at the 5’UTR region and included 25 nucleotides, with the ATG start codon. For *in vitro* use, both GSTM3 and GSTP1 were dissolved in sterile PBS at pH 7.5 and employed at 640 ng/mL. For *in vivo* assays, 15 days post-inoculation of tumors in mice, six doses of 400 ng were intra-tumoral injected, one every three days [40]. In the day 30, all tumors were collected for further analysis. A scrambled morpholino was used as a control in both assays *in vitro* and *in vivo*, at the same concentration. The morpholino sequences employed in these assays were as follows: Morpholino anti-GSTM3 5′-TAG ACG ACT CGC ACG ACA TGG TGA C-3′; Morpholino anti-GSTP1 5′-AAT AGA CCA CGG TGT AGG GCG GCA T-3′; Morpholino Control 5′-TAC GGC GGT ACA GCA CTC AGT TGA T-3′.

### Immunohistochemical staining of tumors generated from CC cell lines

For immunohistochemistry (IHC) analyses; tumors were cut (4 μm thickness) with a cryostat and fixed with 4% paraformaldehyde (Sigma -Aldrich) for 1 h at 37° C. Tissue slides were washed three times with PBS and then blocked with 10% adult bovine serum (Microlab) for 1 h The samples were permeabilized with 0.2% Triton X-100, and tissue sections were individually incubated overnight at 4° C with the following primary antibodies, as described above: anti-NF-κB p65 (phospho-Ser 536), anti-p-JNK, anti-p-ERK and p-p38 from Santa Cruz Biotechnology; anti-GSTM3 and anti-GSTP1 (1:100, Abcam). After several washes with PBS, the tissue sections were then incubated for 1 h at 37° C with the appropriate secondary antibodies; anti-mouse TRITC (1:50, Jackson Immunoresearch), anti-rabbit-FITC (1:100, ThermoFisher), anti-goat Alexa 647 (1:100, ThermoFisher), and anti-mouse-FITC (1:100, ThermoScientific). The negative controls included tissue sections incubated without antibodies as a control for autofluorescence and tissue sections incubated with only the respective secondary antibodies for each condition. The tissue sections were subsequently stained with DAPI (1:50,000) for 5 min. Finally, tumor tissues were mounted with VectaShield mounting medium (H-1000, Vector Laboratories) to preserve immunofluorescence. The immunofluorescence analysis was performed using an LMS 700 Microscope and the accompanying microscope software (ZEN 2012, Carl Zeiss). The presence or absence of the label corresponding to proteins was determined through tile scan analysis (5 × 5 wides in the X, Y planes), combined with Z-stack analysis of three optical slides 0.9 μm apart.

### Immunohistochemical staining of CC tumors

All cases were reviewed by three pathologists, two from of Department of Pathology of INCan and one from the Department of Pathology at *INCMSZ*. The medical records were reviewed, considering the patients previous medical history. All cases were subjected an immunohistochemical analysis using anti-GSTM3 (Abcam, ab67530, 1: 1,000) and, anti-GSTP1 (Abcam, ab53943, 1: 1,000) antibodies. The paraffin blocks were sampled at a 5 µm tissue thickness and were produced in duplicate for each slide. The analysis was performed on an automated immunostainer (Ventana Medical Systems) according to the supplier’s specifications. Three parts of the tumor were assessed separately in each sample, as was the presence of staining in tumor cells. In this study, we analyzed the percentage of the region of interest (ROI) stained by the antibodies, as estimated using the CellSens software (Olympus). The samples were separated into two groups to assess the association of protein expression with patient survival: weak (ROI) for GSTM3 and moderate (ROI) for GSTP1 (W-M); and (MH-H) moderate/high (ROI) for GSTM3 and high (ROI) for GSTP1 [41, 42]. Kaplan-Meier survival curves were employed for this analysis using XLSTAT, with the Greenwood CI and a significance level of 95%.

### Statistical analysis

The experiments were repeated three times, and the results were represented as the mean and standard deviation (SD). Statistical analysis was made using two-tailed Student’s *t*-test. A *p*-value < 0.05 was considered statistically significant.

## SUPPLEMENTARY MATERIALS FIGURES AND TABLES
















