# Supplementary material for: GSTM3 and GSTP1: novel players driving tumor progression in cervical cancer
Source: Oncotarget. 2018 Apr 24;9(31):21696–714. doi: 10.18632/oncotarget.24796 (PMC5955133; doi:10.18632/oncotarget.24796)
Supplement: Supplementary file 1 [file oncotarget-09-21696-s001.pdf]

GSTM3 and GSTP1: novel players driving tumor progression in cervical cancer

SUPPLEMENTARY MATERIALS

Supplementary Material and Methods of Table 10

The synthetic GSTM3 gene was designed according to S. cerevisiae codon usage with the Synthetic Gene Designer program (Wu G *et al*. 2005). The 60 to 80 nucleotide oligos were dissolved at a final concentration of 25 µM, and melting temperature (Tm) was adjusted to approximately 60° C (Supplementary Table 10). Overlapping results for oligo-sets following the two-step PCR method were used for the construction of synthetic gene (Dillon PJ. & CA Rosen. 1990). AccuPrime Pfx DNA polymerase (Invitrogen) was added to the first round of PCR, using an annealing temperature of 60° C, and sequential elongation times of 30 seconds up to 2 min were used for 3–5 cycles. A total of 2 µL of the products was employed as a template for the second PCR using primers with restriction sites flanking the full-length product. The PCR products were cut with the HindIII and BamHI enzymes (Fermentas) and inserted into the pYES2 plasmid. The resulting expression plasmids were replicated in E. coli DH5α and then in S. cerevisiae Σ1278B (MATα ura3) strain. The cells were routinely grown in YEPD medium, and yeast transformation with the expression plasmids was performed as described in the user manual (version K) of the pYES2 kit (Invitrogen). The transformed yeast cells were selected on minimal plates containing 0.67% YND (Difco, BD) with amino acids (Leu-free), 2% dextrose and 2% agar. The induction of recombinant protein was performed in 150mL flasks with 50 mL of induction medium (0.67% YNB with amino acids (Leu-free), 2% L-galactose and 2% raffinose). We inoculated the cultures with approximately 5 × 105 yeast cells/mL in each flask. After 72 hours of incubation at 30° C with constant shaking (200 rpm), the cells were collected, flash-frozen and stored at –80° C. The induced yeast cells were washed with 50 mL ddH2O three times and resuspended in 1 mL of 50 mM Kpi buffer (7.0 pH), with 5% glycerol and a protease inhibitor cocktail (Complete tablet, Roche).

REFERENCE OF SUPPLEMENTARY TABLE 10

1. Dillon PJ, Rosen CA. A rapid method for the construction of synthetic genes using the polymerase chain reaction. Biotechniques. 1990; 9:298, 300.
2. Wu G, Bashir-Bello N, Freeland S. The synthetic gene designer: A flexible Web platform to explore sequence space of synthetic genes for heterologous expression. 2005 IEEE Comput Syst Bioinforma Conf Work Poster Abstr. 2005. page 258–9.

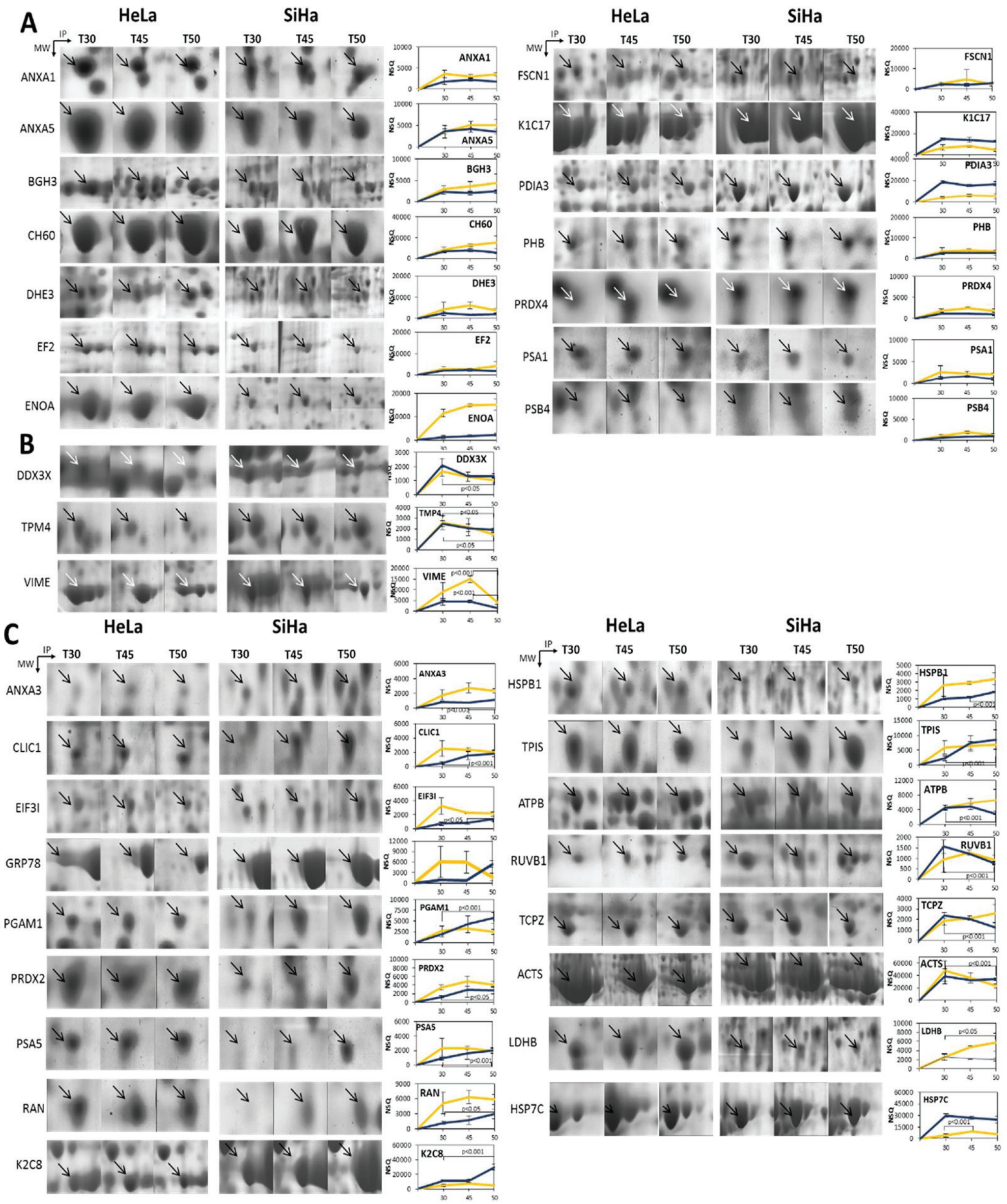

Supplementary Figure 1: Shared proteins in HeLa and SiHa tumors. Representative image of each protein by different day (30, 45 and 50). (**A**) 14 with constant expression in HeLa and SiHa tumors. (**B**) Subexpression of 3 proteins across the time in HeLa and SiHa. (**C**) 17 Proteins with different expression between HeLa and SiHa tumors. Spot normalization was employed using total density in gel image, to ensure the independence of the data from experimental variations between gels. Student's *t*-test was performed to assess the significance of differences between differentially expressed proteins.

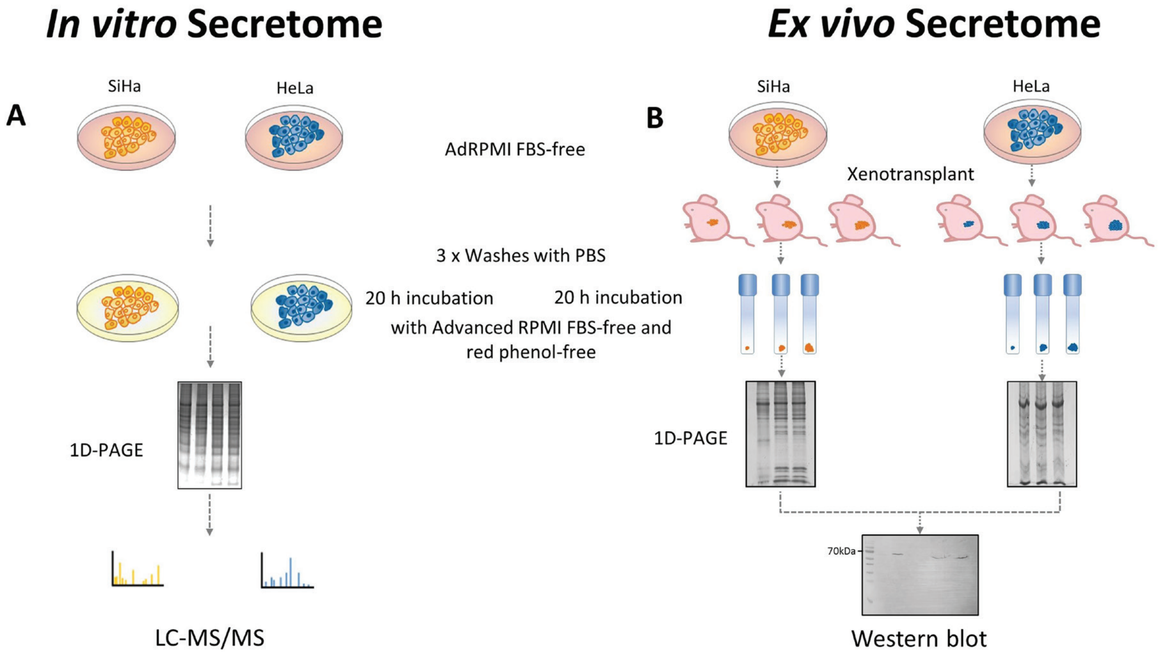

Supplementary Figure 2: Workflows for obtaining *in vivo* or *ex vivo* secreted proteins. Cell lines were cultured in advanced RPMI 1640 (Gibco, Invitrogen) serum-free medium until 70–80 % of confluence was reached in 500 cm[2] Triple Flasks (Nunc Clon). The medium was removed and cells were rinsed three times with 500–600 mL of sterile physiological solution (NaCl 0.9% (w/v)). After washing, fresh RPMI 1640 phenol red-free medium was added (Gibco, Invitrogen) and cells were incubated for 20 hours. Later, the medium was removed and centrifuged at 1,500 g for 5 min. The supernatant was passed through a 0.22 µm pore size PVDF membrane (Millex, Millipore) and stored at –70° C until further use. For secreted proteins from HeLa and SiHa tumors, female Nu/Nu mice (age 4–6 weeks) were inoculated with 107 cells. After 30, 45 and 50 days' post-inoculation, tumors were collected (3 different replicates were used) and washed 3 times with 50 mL of physiological solution to then be incubated 20 hours, with phenol red-free and serum-free RMPI. As we did for we *in vitro* experiments, the medium was removed and centrifuged at 1,500 g for 5 min. The supernatant was passed through a 0.22 µm pore size membrane PVDF (Millex, Millipore) and stored at –70° C until further use. Subsequently, *in vitro* and *ex vivo* secreted proteins were lyophilized and resuspended in 1 mL ultrapure water. Protein isolation was performed through phenol extraction as described in Methods.

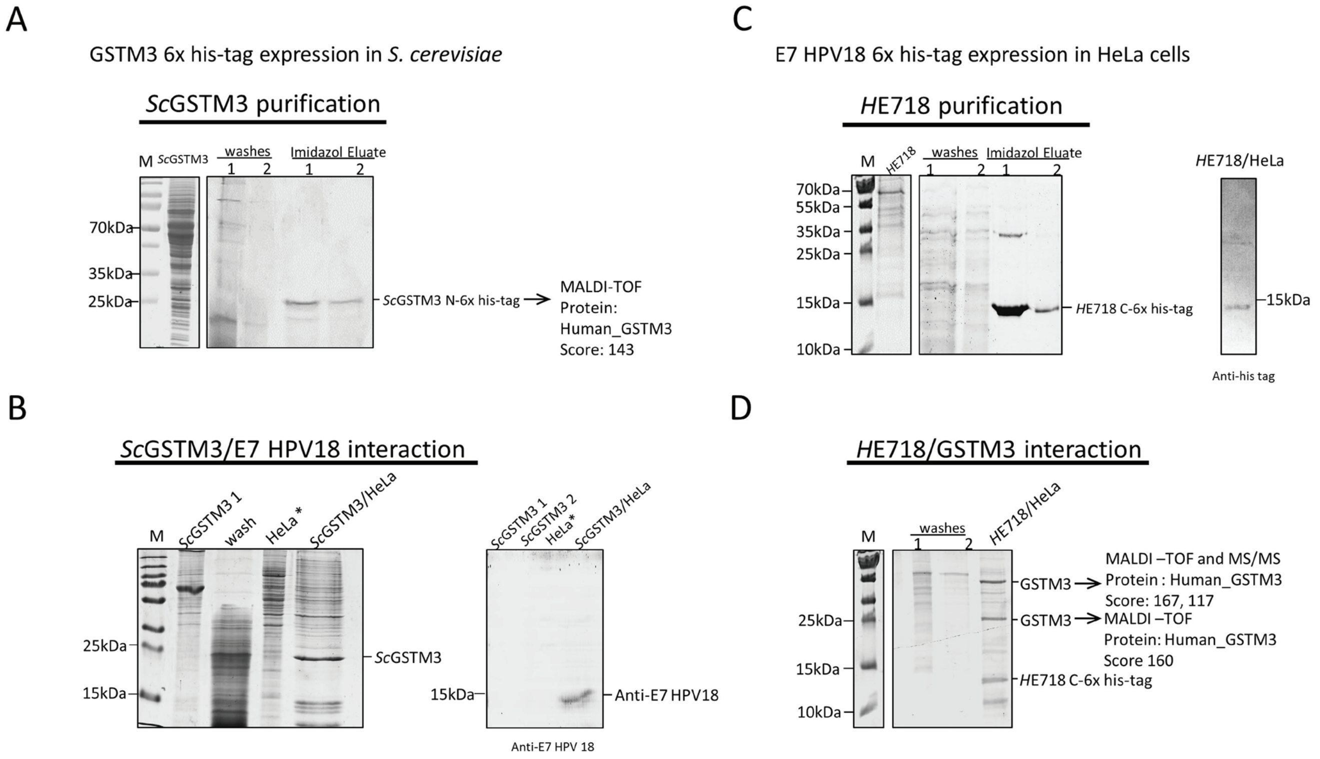

Supplementary Figure 3: Yeast plasmids construction, transformation, and recombinant protein expression. (**A**) Recombinant human protein GSTM3 with a His-tag was expressed in Saccharomyces cerevisiae. The samples were separated on 12% SDS-PAGE. Line 1: Markers, Line 2: ScGSTM3 lysate (GSTM3 N-6x His-Tag on Saccharomyces cerevisiae), Line 3 and 4: purification washes, Line 5 and 6: purified ScGSTM3 with 300 mM of Imidazole. ScGSTM3 was identified through peptide mass fingerprinting using the Mascot program (Matrix Science). (**B**) After capturing the recombinant GSTM3, it was incubated with a protein extract of HeLa cells (HPV18 positive) at 4° C overnight. GSTM3 6xhis-tag protein interactions were analyzed. Line 1: Markers, Line 2 and 3: ScGSTM3 flow throughput, Line 4: HeLa proteins that do not interact with ScGSTM3, Line 5: HeLa proteins interaction with ScGSTM3. HPV18 E7 protein co-eluted with ScGSTM3 and could be identified using western blot (20 µg of protein and a specific antibody). (**C**) Recombinant protein of HPV18 E7 with a His-tag expression in HeLa cell line. The samples were separated on 15% SDS-PAGE. Line 1: Markers, Line 2: HeLa flow throughput, Line 3 and 4: purification washes, Line 5 and 6: purified HeLaE718 with 300mM of Imidazole. HeLaE718 was identified by western blot with specific antibody his-tag. (**D**). After capturing the recombinant HPV18 E7 6x his-tag with Nickel beads, it was incubated to 4° C overnight with HeLa proteins. Line 1: Markers, Line 2 and 3: purification washes, Line 4: HeLa proteins that interact with HE718. GSTM3 protein co-elute with HPV18 E7 protein could be identified using western blot and peptide mass fingerprinting as well as LC-MS/MS (20µg of protein and a specific antibody).

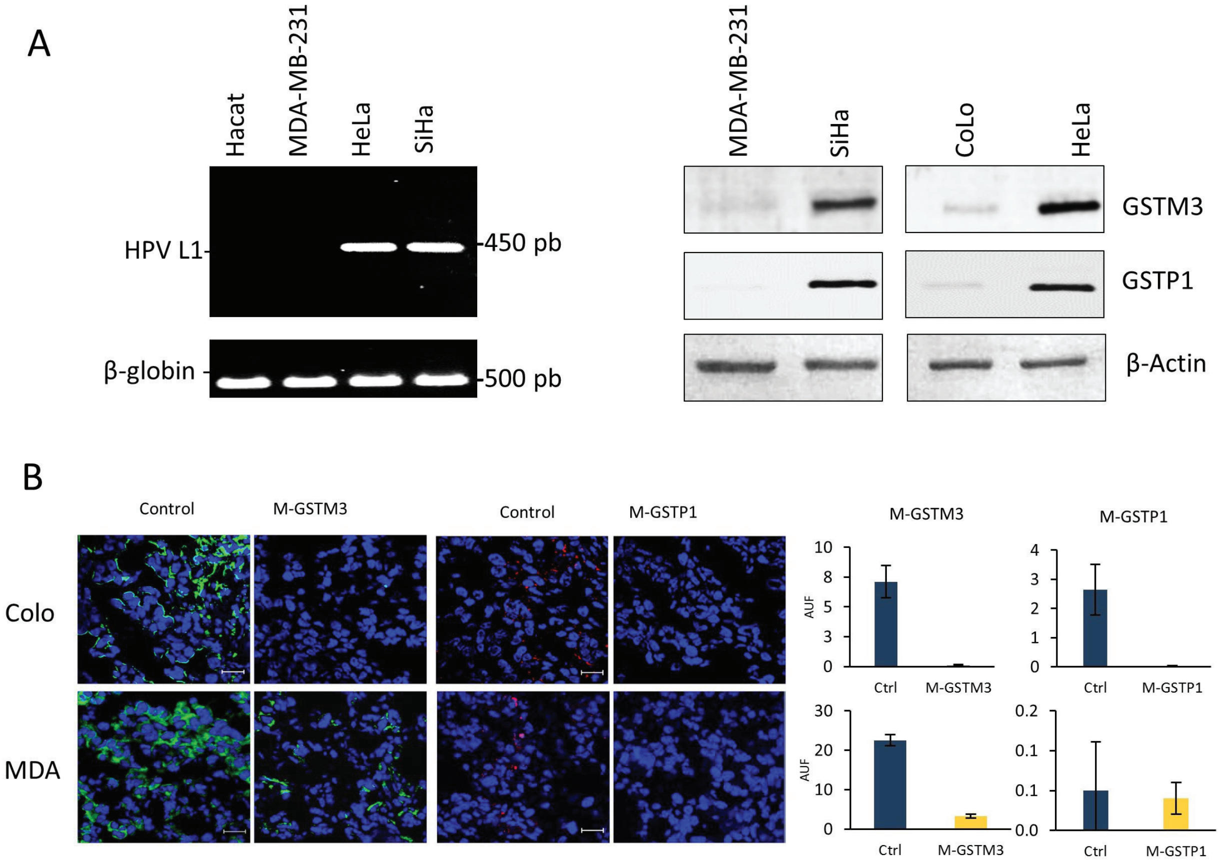

Supplementary Figure 4: (**A**) MDA-MB-231 negative to HPV18 (1) and GSTs (GSTM3 and GSTP1). Electrophoresis gel 1% agarose with PCR products of L1 gene of HPV and β-globin as a control, and western blot of GSTM3 and GSTP1 (**B**) GSTM3 and GSTP1 protein expression in MDA (breast cancer) and COLO (colon cancer) tumors. A negative expression in GSTP1 protein in MDA cell line had no significant changes in tumor growth. COLO cell line showed a decrease in GSTP1 protein expression with morpholino treatment and a decrement of tumor growth of 47.7% with M-GSTM3 treatment.

Depuydt CE. *et al*. Comparison of MY09/11 consensus PCR and type-specific PCRs in the detection of oncogenic HPV types. J Cell Mol Med. 2007;11:881–91.

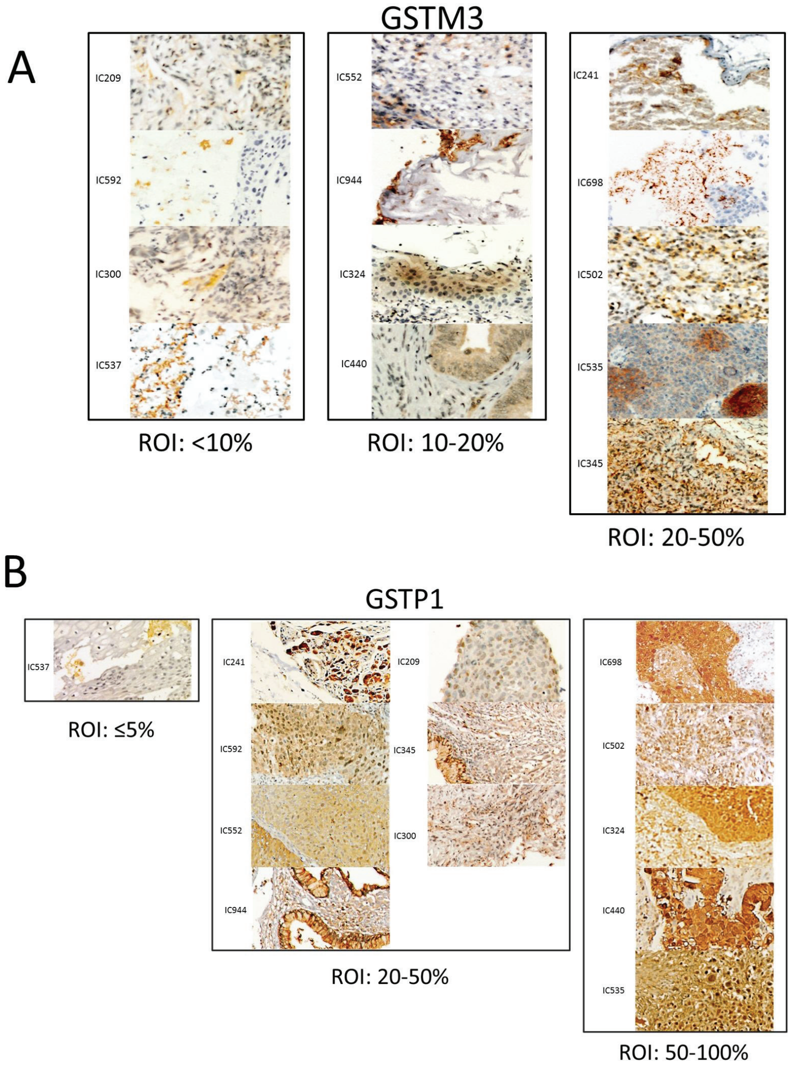

Supplementary Figure 5: GSTM3 and GSTP1 expression in late-stage cervical cancer. Biopsy samples were stained with anti-GSTM3 and anti-GSTP1 antibodies. Three representative microphotographs of each CC tumor biopsy are exhibited and were used for ROI analysis.

Supplementary Table 1:The proteomic profile was obtained from each time and then compared to find differentiated proteins during TP

	**HeLa**	**SiHa**
Time	Spots/CCf	Spots /CCf
T30	824/0.710	765/ 0.731
T45	763/0.723	768/0.836
T50	1012/ 0.724	766/0.756

Total spots by time and correlation coefficient (CCf) was calculated to guarantee representative results.

Supplementary Table 2: Total proteins expressed in HeLa tumors (30, 45 and 50 days). See Supplementary_Table_2

Supplementary Table 3: Total proteins expressed in SiHa tumors (30, 45 and 50 days). See Supplementary_Table_3

Supplementary Table 4: Enrichment analysis of shared proteins in HeLa and SiHa tumors, using GeneCodis: Biological process categories as gene sets. See Supplementary_Table_4

Supplementary Table 5: Reactome pathways of shared proteins in HeLa and SiHa tumors. See Supplementary_Table_5

Supplementary Table 6: Relative levels of MAPK, NFkB, TRAF6 and TLR4

HeLa/ protein	Time	Means (Relative to Actin)	SD	SiHa/ protein	Time	Means (Relative to Actin)	SD
ERK	30	0.53	0.18	ERK	30	0.32	0.01
	45	0.54	0.14		45	0.33	0.05
	50	0.55	0.08		50	0.26	0.01
pERK	30	0.13	0.03	pERK	30	0.11	0.01
	45	0.06	0.02		45	0.13	0.02
	50	0.09	0.01		50	0.17	0.05
JNK	30	0.17	0.08	JNK	30	0.11	0.02
	45	0.17	0.01		45	0.12	0.02
	50	0.24	0.06		50	0.15	0.04
pJNK	30	0.04	0.02	pJNK	30	0.01	0.00
	45	0.03	0.02		45	0.03	0.02
	50	0.04	0.01		50	0.01	0.00
p38	30	0.32	0.08	p38	30	0.52	0.06
	45	0.36	0.06		45	0.42	0.03
	50	0.34	0.10		50	0.41	0.06
pp38	30	0.04	0.02	pp38	30	0.01	0.00
	45	0.06	0.01		45	0.01	0.00
	50	0.03	0.01		50	0.01	0.00
NF-kB	30	0.34	0.01	NF-kB	30	0.39	0.02
	45	0.37	0.01		45	0.26	0.03
	50	0.22	0.04		50	0.43	0.01
pNF-kB	30	0.13	0.04	pNF-kB	30	0.11	0.02
	45	0.19	0.04		45	0.14	0.01
	50	0.15	0.03		50	0.11	0.02
IKBα	30	0.27	0.01	IKBα	30	0.20	0.00
	45	0.25	0.01		45	0.22	0.02
	50	0.24	0.01		50	0.20	0.02
p-IKBα	30	0.00	0.00	p-IKBα	30	0.00	0.00
	45	0.00	0.00		45	0.00	0.00
	50	0.08	0.01		50	0.00	0.00
TRAF6	30	0.14	0.02	TRAF6	30	0.00	0.00
	45	0.25	0.06		45	0.00	0.00
	50	0.25	0.09		50	0.00	0.00
TRL4	30	0.06	0.02	TRL4	30	0.93	0.05
	45	0.15	0.02		45	0.24	0.04
	50	0.46	0.05		50	0.71	0.08

Supplementary Table 7: Secreted proteins in HeLa cells. 432 proteins identificated. See Supplementary_Table_7

Supplementary Table 8: Secreted proteins in SiHa cells. See Supplementary_Table_8

Supplementary Table 9: Distances in 2 and 3 Ångströms (Å) between the GSTM3 and E7 HPV18

		Protein		
**Distance**	**HPV 18 E7**		**GSTM3**	
**2Å**	**Aminoacid**	**Position**	**Aminoacid**	**Position**
	HIS	51	ASP	102
	TYR	52	ARG	82
	ASN	53	ASN	106
	PHE	57	GLN	76
	MET	84	LYS	73
	GLU	85	ASP/PHE	60/61
	LEU	87	PHE	61
		**Protein**		
**Distance**	**HPV 18 E7**		**GSTM3**	
3Å	Aminoacid	Position	Aminoacid	Position
	ALA	50	GLU	95
	HIS	51	ASP	102
	TYR	52	ARG/GLU	82/105
	ASN	53	GLU/ASN	105/106
	PHE	57	GLN	76
	ARG	66	ASN	106
	HIS/LYS	63/71	LYS	98
	LEU	83	PHE	61
	MET	84	LYS	73
	GLY	85	ASP/PHE	60/61
	THR	86	PHE	61
	LEU	87	PHE	61
	GLY	88	PHE	61

Supplementary Table 10: List of primers used to generate recombinant GSTM3 protein. See Supplementary_Table_10

Supplementary Table 11: Primers used for amplification and cloning of the HPV18 E7 gene

Name	Sequence 5′ > 3′	Comments
E718-1	ATG CAT GGA CCT AAG GCA ACC ATT	
E718-2*	CTG CTG GGA TGC ACA CCA	
E7-18-Hind III	ATA CAA **AAG CTT** ATG CAT GGA CCT AA	This oligo contains the HindIII restriction site (bold), and initial sequence of HPV 18 (bold).
E7HPV18-his*	**GAT GGT GAT GAT** GCT GCT GG	This oligo contains a fragment 6x histidine sequence (bold).
Univ His-Tag BamH I*	TAC GTG **GAT** CCT **AGT GGT GAT GGT G**	This oligo contains the BamHI restriction site (bold) and fragment of 6x histidine's sequence (bold and underlined)

30 cycles of a standard PCR protocol were performed using the following program: 94° C for 1 min, 60° C for 45 seconds and 72° C for 2 min.

Supplementary Table 12: ROI of GSTs proteins of CC patients

Protein	ID	Mean Sum of stain (Area) (μm²)	Mean Sum of no stain (Area) (μm²)	Total Area of cell (μm²)	Mean of AREA FRACTION ROI %	Classification	Protein	Mean Sum of stain (Area) (μm²)	Mean Sum of no stain (Area) (μm²)	Total Area of cell	Mean of AREA FRACTION ROI %	Classification
GSTM3	502	3,11,243.79	7,35,599.42	10,46,843.21	29.73	High	GSTP1	8,14,269.62	4,42,021.41	12,56,291.03	64.8	High
	300	36,571.91	5,27,141.79	5,63,713.70	6.49	Weak		5,36,105.53	5,66,210.48	11,02,316.01	48.6	Moderated
	698	67,007.39	1,77,911.74	2,44,919.13	27.36	High		5,11,678.95	4,88,372.19	10,00,051.14	51.2	High
	324	1,13,134.55	5,80,128.64	6,93,263.20	16.32	Moderated		8,95,090.00	4,48,948.52	13,44,038.52	66.6	High
	345	4,50,109.91	8,38,099.43	12,88,209.34	34.94	High		5,77,972.52	6,86,381.85	12,64,354.38	45.7	Moderated
	944	1,26,711.35	6,55,983.41	7,82,694.77	16.19	Moderated		5,16,617.31	6,96,180.99	12,12,798.30	42.6	Moderated
	535	2,53,528.44	5,64,860.42	8,18,388.86	30.98	High		8,69,484.57	3,80,402.05	12,49,886.62	69.6	High
	241	2,36,100.04	6,39,148.15	8,75,248.19	26.98	High		2,32,216.69	7,61,975.89	9,94,192.58	23.4	Moderated
	209	22,949.68	4,84,613.05	5,07,562.73	4.52	Weak		2,65,209.56	3,55,823.84	6,21,033.40	42.7	Moderated
	592	39,824.78	8,30,366.61	8,70,191.39	4.58	Weak		2,21,299.66	4,90,337.19	7,11,636.84	31.1	Moderated
	440	2,00,506.09	9,85,348.59	11,85,854.68	16.91	Moderated		8,77,113.35	4,20,341.69	12,97,455.04	67.6	High
	552	1,13,462.72	6,17,323.87	7,30,786.59	15.53	Moderated		2,40,094.59	3,45,836.90	5,85,931.49	41.0	Moderated
	537	72,269.62	6,80,970.07	7,53,239.69	9.59	Weak		37,216.89	7,08,046.98	7,45,263.87	5.0	weak
